# SPiKe-Med: a hybrid spiking-CNN-transformer framework for energy-efficient brain tumor MRI segmentation

**DOI:** 10.3389/frai.2026.1852940

**Published:** 2026-09-09

**Authors:** M. Sujatha, B. Aarthi

**Affiliations:** Vellore Institute of Technology - Chennai Campus, Chennai, India

**Keywords:** brain tumor, deep learning, Magnetic Resonance Imaging, medical image analysis, transformer networks, spiking neural network, Neuromorphic computing

## Abstract

Early disease detection is crucial for any disease it helps in better treatment and curing. For this purpose, several Artificial Intelligence (AI)-assisted research studies have been developed and tested for medical image processing. Although traditional Deep Learning (DL) models aid in disease detection, computational complexity and energy-intensive challenges limit the performance. For this reason, this research proposes SPiKe-Med, an efficient, accurate and neuromorphic-inspired solution by incorporating Spiking Neural Networks (SNNs) into state-of-the-art Convolutional Neural Network (CNN) and transformer backbones architectures. Stage 1 uses standardized preprocessing, N4 bias-field correction, isotropic resampling, *z*-score intensity normalization, and modality-specific augmentations. Stage 2 proposes Swin Unet-based Transformer-Network (SUT-Net), a high-performance dense segmentation backbone configured using benchmark Swin Transformer and UNET- Transformers (UNETR) for long-range context. Stage 3 develops the proposed SPiKe-Med by training multi-scale feature maps into spike trains with a learned encoder (temporal encoding) and feeds them to a spiking refinement network of Leaky Integrate-and-Fire (LIF) neurons trained as reconfigurable with SpikingJelly. Stage 4 trains the proposed SPiKe-Med on a two-track schedule: end-to-end surrogate-gradient optimization of SNN components (surrogate derivatives) and selective ANN to SNN porting of pretrained CNN encoder weights to ultra-low-latency inference. Loss functions combine a Dice, focal and temporal consistency term, which is optimized by temperature scaling. The proposed SPiKe-Med is tested with multiple benchmark datasets for different modalities like MRI and CT using Dice, Hausdorff distance, spike-rate and estimated energy per inference.

## 1 Introduction

Medical imaging is an important aspect in the current healthcare, especially in early detection, diagnosis and monitoring of illnesses (Zhu et al., 2023). Brain tumors are one of the most difficult disorders because of their heterogeneity, complicated morphology, and rapid evolution, which greatly influence patient prognosis when they are not identified and treated in time (Aggarwal et al., 2023). MRI has become the imaging modality of choice, including high-resolution imaging of the soft tissues and allowing the detailed evaluation of the tumor boundaries, volume and the surrounding structures (Tandel et al., 2022). Manual segmentation of MRI scans to delineate tumors is, however, labor-intensive, and reliable segmentation is therefore a necessity. AI and DL methods, in this regard, have shown a lot of potential, using hierarchical feature learning to automate segmentation with higher accuracy, consistency, and speed than conventional approaches (Chukwujindu et al., 2024). These techniques minimize the reliance on manual intervention and offer a reliable structure of clinical decision-making by capturing complex spatial patterns and variations in intensity (Younis et al., 2022; Montaha et al., 2023).

Over the years, several techniques have been explored for automated brain tumor segmentation. Classical image processing techniques were first used in order to identify tumor areas using intensity or edge data (Vankdothu and Hameed, 2022). Statistical and machine learning methods then tried to classify the type of tissue based on handcrafted features of intensity, texture or shape descriptors (Renugadevi et al., 2023). The advent of DL made the use of CNNs very popular because they automatically learn hierarchical representations, which allow them to perform better pixel-level segmentation (Ullah et al., 2023). This was subsequently followed by Recurrent Neural Networks (RNNs) and attention-based systems that learn spatial or temporal dependencies in multi-slice MRI scans to improve context-awareness (Amarneni and Valarmathi, 2024; Mushtaq et al., 2022). Also, lightweight network architecture and energy-efficient architectures have been considered to enable them to be deployed in low-resource or real-time environments (Wu et al., 2023).

Even with these developments, the current methods have a number of shortcomings. The conventional CNN and attention-based networks are precise but are computationally expensive and energy-intensive, and are not utilized in real-time or resource-limited clinical settings (Nizamani et al., 2023; Balamurugan and Gnanamanoharan, 2023). Lightweight or neuromorphic approaches are energy-efficient, but tend to reduce segmentation accuracy and do not effectively model long-range spatial information (Biratu et al., 2021). To address these issues, the current study introduces SPiKe-Med, a hybrid architecture that integrates the feature extraction power of deep networks with the power efficiency of SNNs. SPiKe-Med allows for achieving high clinical reliability with accurate, low-latency, and energy-efficient brain tumor segmentation by combining multi-scale feature learning, temporal encoding, and a spiking refinement network, which bridges the critical gaps of current methods. The contribution of the research is given below:

- SPiKe-Med framework is proposed by integrating CNN, Swin Transformer, and SNN architectures for brain tumor segmentation.
- Temporal spike encoding with LIF neurons is introduced to achieve energy-efficient and low-latency neuromorphic processing.
- The proposed framework jointly achieves higher segmentation performance, lower energy consumption, reduced spike activity, and faster inference through ANN-to-SNN conversion and spike-driven refinement.

## 2 Literature review

In 2023, Sangui et al., proposed a 3D U-Net architecture to identify brain tumor based on MRI scans (Sangui et al., 2023). The model used an encoder-decoder architecture consisting of convolutional, pooling and up-sampling layers to extract spatial and contextual information. It was done by preprocessing volumetric MRI data and training the network to be able to segment the tumor. The technique enhanced the localization of tumor boundaries and minimized human interference. The limitations are that it has great computational demands and that it does not handle tumors with non-homogeneous shape and size.

In 2023, Rajendran et al., suggested an automated DL model to segment brain lesions in MRI images (Rajendran et al., 2023). The approach used a CNN to acquire hierarchical features and improve tumor regions. This was done by preprocessing MRI scans, training the network on annotated datasets, and doing pixel-level segmentation. The method proved to be more accurate and less manual labor intensive than the conventional methods.

In 2022, Guan et al., developed 3D AGSE-VNet, a model to automatically segment brain tumors in MRI images (Guan et al., 2022). The model was used to combine 3D convolutional layers with attention-directed spatial and channel enhancement modules to learn detailed tumor features. It involved preprocessing of MRI scans, inputting volumetric data into the network, and doing accurate voxel-level segmentation. Limitations, however, were greater computational complexity and possible performance degradation in highly heterogeneous tumor regions.

In 2025, Akan et al., suggested a multimodal optimization system for brain MRI segmentation, which combines several different imaging modalities to improve the extraction of tumor features (Akan et al., 2025). It was done by preprocessing MRI data, optimizing it, and segmenting it at a voxel level. This method enhanced segmentation accuracy and strength among tumor types. The weaknesses were a higher level of computational complexity and difficulty with highly heterogeneous tumor regions.

In 2025, Tiwary et al., proposed a DL model based on EfficientNet and U-Net to segment the MRI brain diseases (Tiwary et al., 2025). This model used EfficientNet as an encoder to obtain rich features, and then a U-Net decoder to obtain accurate tumor delineation. This involved MRI preprocessing, network training and pixel-level segmentation. The method increased accuracy and minimized manual annotation requirements, but was computationally intensive and had difficulty with irregular tumor shapes.

In 2025, Qiu et al., suggested a framework, QCResUNet, to predict the quality of subject-level and voxel-level segmentation in brain MRI (Qiu et al., 2025). The model was a combination of a residual U-Net architecture and quality assessment modules to predict the reliability of segmentation. It involved preprocessing of MRI images, network training and assessment of segmentation and quality measures. The method enhanced trust in the outputs of the segmentation, but the disadvantages were the complexity of the models and the reliance on large annotated datasets.

In 2021, Rehman et al., suggested BrainSeg-Net, a network to segment brain tumor MRI (Rehman et al., 2021). The model has used convolutional layers with skip connections to simultaneously represent local and global tumor features. This was done by preprocessing MRI scans, training the network on annotated datasets, and performing pixel-level segmentation. Nevertheless, drawbacks were increased computational needs and decreased performance on irregularly shaped or low-contrast tumors.

In 2023, Peng and Sun suggested AD-Net, which combined an attention-based encoder-decoder network to obtain complementary characteristics between different MRI modalities (Peng and Sun, 2023). This involved preprocessing of multimodal MRI scans, network training and voxel-level segmentation. This method improved precision in tumor delimitation and tumor classification. Disadvantages were more complex computations and the difficulty in cutting irregularly-shaped or low-contrast tumors.

In 2023, Raza et al., presented dResU-Net, a multimodal MRI 3D deep residual U-Net brain tumor segmentation model (Raza et al., 2023). The model was used to jointly learn residual connections with 3D convolutional layers to learn complex spatial features across modalities. It included preprocessing volumetric MRI data, network training and voxel-level segmentation. This method enhanced better segmentation and minimized human work. Disadvantages were high computing requirements and difficulties in the segmentation of highly irregular tumor structures.

In 2022, Iqbal et al., presented a super-pixel-based method of automatic segmentation (Iqbal et al., 2022). The algorithm separated MRI scans into super-pixels to simplify the computations and used feature extraction to localize the tumor accurately. This involved preprocessing of MRI slices, superpixel generation and classification of tumor regions. This method saved time on processing and was not very inaccurate. Limitations included reduced performance on tumors with complicated edges or changing intensities.

In 2023, Gómez-Guzmán et al., presented an automated brain tumor classification approach using MRI images based on deep learning (Gómez-Guzmán et al., 2023). This research involved 7,023 MRI images, which were categorized into glioma, meningioma, pituitary tumor, and normal brain classes. Several different types of CNN models were investigated, and the most successful one had an accuracy of 97.12%. The findings confirmed the ability of DL algorithms to be used in brain tumors' detection. But the paper focused only on classification results without further studies of tumor segmentation and model robustness, and explainability.

In 2025, Gómez-Guzmán et al., proposed a DL architecture for multi-class brain tumor classification of multi-class using MRI images with the aid of CNN and transformer architectures (Gómez-Guzmán et al., 2025). The research involved the use of Brain Tumor MRI Msoud dataset, which had 7,023 MRI images categorized as glioma, meningioma, pituitary tumor, and no tumor. Techniques such as transfer learning, data augmentation, and stratified five-fold cross-validation were used to enhance the performance of the model. The most effective model attained high accuracy and showed efficiency in classification and generalization. However, the research mainly focused on classification and edge deployment.

In 2026, Gómez-Guzmán et al., explored the problem of glioma classification from MRI scans using novel DL methods that relied on convolutional neural networks and vision transformers (Gómez-Guzmán et al., 2026). In their research, the BraTS 2019 dataset was used, which comprised of 2,185 MRI scans belonging to either HGG (high-grade glioma) or LGG (low-grade glioma). Cross-validation of three-folds along with various ratios of training data was used to evaluate the models in conditions of limited training data. The most efficient one was found to have an accuracy of 99.401%. However, glioma classification was considered in the study without exploring multi-tumor segmentation.

### 2.1 Problem statement

Although the automated brain tumor segmentation has achieved a lot of progress, the current solutions still have major challenges that hamper their practical use. The computational requirements and energy-consuming processing make it difficult to scale and deploy in real-time in clinical environments. Furthermore, it is also a continuing challenge to precisely detect tumors with irregular shapes, heterogeneous structures, or low-contrast areas, which frequently leads to incomplete or inaccurate delineation. Besides lowering reliability, these restrictions also increase the need to rely on manual intervention, which impacts the overall diagnostic efficiency. This has resulted in the requirement of a strong, energy-efficient and precise segmentation scheme that supports a wide range of tumor properties and provides quick and reliable results in clinical practice. Table 1 presents a comparative summary of recent brain tumor segmentation techniques, findings and limitations.

**Table 1:** Summary of recent DL techniques for brain tumor segmentation.

References	Technique used	Key findings	Limitations
Sangui et al. (2023)	3D U-Net	Better localization of tumor boundaries, less human intervention	Large computation requirements, non-homogeneous tumor shapes and sizes are challenging
Rajendran et al. (2023)	CNN-based automated segmentation	Better segmentation, less manual work	Irregular tumor shapes lead to poor performance
Guan et al. (2022)	3D AGSE-VNet with attention-guided spatial & channel enhancement	More detailed voxel-based segmentation, better tumor features	Low calculability, decreased action against non-homogenous tumors
Akan et al. (2025)	Multimodal optimization-based segmentation	Improved accuracy of segmentation of tumors	Complex computing, inability to work with highly heterogeneous tumor regions
Tiwary et al. (2025)	EfficientNet-enhanced U-Net	Better tumor delineation, less manual annotation	Computationally expensive, does not deal with irregular tumor shapes
Qiu et al. (2025)	QCResUNet	Anticipated segmentation accuracy, increased confidence in outputs	Large model complexity needs large annotated datasets
Rehman et al. (2021)	BrainSeg-Net	Local and global tumor features are captured, and segmentation accuracy is enhanced	Greater computing requirements, reduced irregular or low-contrast tumor performance
Peng and Sun (2023)	AD-Net	Improved delimitation and classification of tumors	Complicated calculations, inability to work with irregular tumors or low contrast
Raza et al. (2023)	dResU-Net	Better segmentation, less manual processing	Complex computing needs, inability to deal with highly irregular tumor forms
Iqbal et al. (2022)	Superpixel-based segmentation	Less time for processing localization of tumors	Poor performance on complex-edge or variable intensity tumors
Gómez-Guzmán et al. (2023)	CNN-based MRI tumor classification	Effective tumor classification using CNN models	Lacked segmentation and explainability analysis
Gómez-Guzmán et al. (2025)	CNN and transformer-based transfer learning	Improved multi-class tumor classification with advanced architectures	Limited clinical validation and interpretability
Gómez-Guzmán et al. (2026)	CNN and vision transformer-based glioma classification	Achieved reliable glioma classification with limited data	Limited to glioma classification and lacked comprehensive segmentation

## 3 Proposed methodology

**Figure 1:**
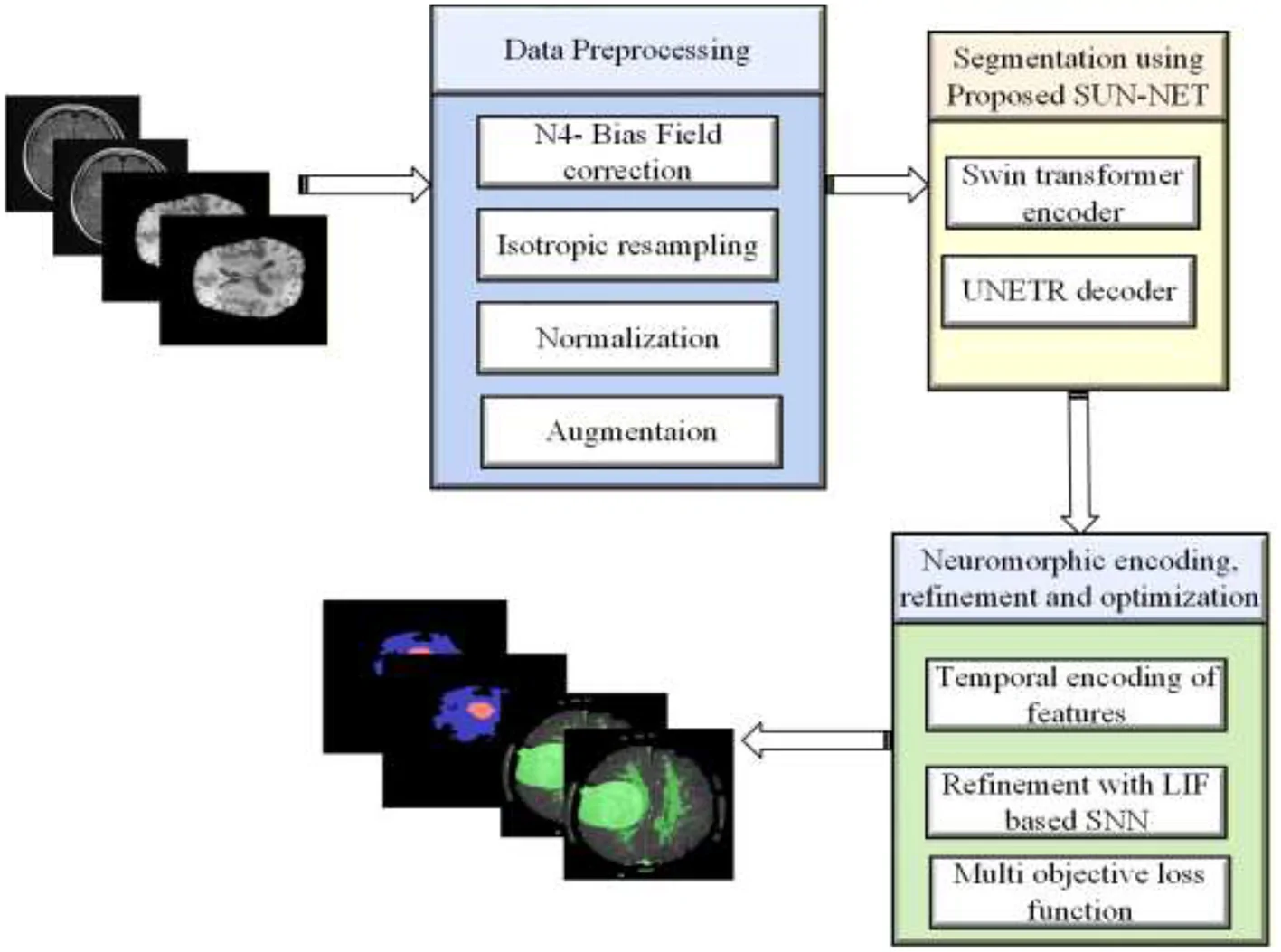
Conceptual architecture of the proposed model.

The SPiKe-Med framework proposes a hybrid system for brain tumor segmentation of MRI and CT images, incorporating advanced feature extraction with neuromorphic-inspired processing to obtain precise and energy-efficient outputs. The framework operates through a multi-step pipeline, where the first stage involves extensive preprocessing. This includes N4 bias-field correction, isotropic resampling, *z*-score intensity normalization, and modality-specific augmentations to normalize and enhance the input images. This is followed by a thick segmentation backbone, which is used to obtain multi-scale feature maps. The resulting feature maps are then converted into temporal spike trains by a learned encoding mechanism, and refined segmentation is performed by an SNN made of LIF neurons. The training process is based on a two-track approach, which involves end-to-end spiking component surrogate-gradient optimization, with selective transfer of existing weights between traditional networks to obtain ultra-fast inference. A set of Dice, focal, and temporal consistency loss functions optimized using temperature scaling is used as well to improve segmentation accuracy and stability. Figure 1 illustrates the conceptual architecture of the proposed model. The complete workflow of the proposed framework is summarized in Algorithm 1.

Algorithm 1 **Proposed SPiKe-Med Framework for Brain Tumor Segmentation.**

**Input:** Multimodal MRI/CT brain images *I*
**Output:** Segmented tumor mask *S*
Step:1   Acquire multimodal brain MRI and CT images from the dataset.
Step:2   Perform preprocessing, including bias-field correction, intensity normalization, resizing, and data augmentation.
Step:3   Extract hierarchical spatial features using the CNN-based feature extraction module.
Step:4   Feed the extracted features into the Swin Transformer-based SUT-Net backbone for global contextual learning and long-range dependency modeling.
Step:5   Apply spike-based temporal encoding to convert continuous activations into temporal spike representations.
Step:6   Process the temporal spike sequences using Leaky Integrate-and-Fire (LIF) neurons for neuromorphic feature refinement.
Step:7   Apply attention-residual refinement to enhance tumor-boundary localization and contextual consistency.
Step:8   Optimize the framework using a composite loss function consisting of Dice loss, Focal loss, and Temporal Consistency loss.
Step:9   Generate the final segmented tumor mask *S*.
Step:10   Evaluate segmentation performance using quantitative metrics and computational-efficiency measures.

The methodology guarantees an extensive feature representation, efficient computation and dependable functionality in the context of heterogeneous tumor structures, rendering it appropriate to be applied in real clinical settings. SPiKe-Med is a scalable solution, bridging high-performance DL and low-energy spiking computation, applicable to real-time medical imaging applications.

### 3.1 Dataset description

Two publicly available brain imaging datasets were used for evaluation of the proposed model. The BraTS20 Training and Validation dataset consists of 369 patient cases with four different imaging modalities such as T1, T1ce, T2, and FLAIR and expert annotations for tumor segmentation masks. Each patient case includes approximately 155 axial slices resulting in about 57,195 MRI slices without preprocessing. Non-informative slices were eliminated, and the rest of the images were used for training the models. Patient-level splitting was utilized to create training and validation subsets to ensure that the images of one patient do not belong to different subsets. The Brain Tumor CT Images Binary Classification dataset is composed of 3,264 CT images, where 1,641 are tumor images, and 1,623 are normal. Each image has an image-level label of tumor presence/absence. Image resizing and normalization are done before training models. Since there were no patient identifiers, image-level splitting was done carefully to reduce the risk of data leakage. Duplicate images were excluded, and preprocessing was done after splitting.

### 3.2 Preprocessing

Preprocessing is necessary to remove artifacts caused by the scanner, correct intensity variations, and standardize the spatial resolution, ensuring the input data is clean, uniform, and suitable for training the model reliably and segmenting it correctly. The suggested SPiKe-Med system uses a multi-step preprocessing pipeline as explained below.

#### 3.2.1 N4 bias-field correction

The magnetic field non-uniformity that is found in MRI images tends to create intensity inhomogeneity, and this negatively impacts segmentation accuracy. The N4 bias-field correction algorithm is used to fix this. This is expressed in Equation 1:


$$\begin{array}{l} I_{obs}\left(v\right)=I_{true}\left(v\right).\;B\left(v\right) \end{array}$$ (1)

where *I*_*obs*_(*v*) shows the observed intensity, *v* represents voxel, *I*_*true*_(*v*) is used to represent the true tissue identity and *B*(*v*) represents the bias field. The estimated bias field is eliminated to get the corrected image. The corrected image is recovered by voxel-wise division of the observed signal by the estimated bias field, as mentioned in Equation 2, where *I*_*corrected*_(*v*) denotes bias-corrected intensity and $\hat{B}\left(v\right)$ denotes the estimated bias field.


$$\begin{array}{l} I_{corrected}\left(v\right)=\frac{I_{obs}\left(v\right)}{\hat{B}\left(v\right)} \end{array}$$ (2)

This step equalizes the intensity fluctuations and improves the tissue contrast to increase the accuracy of the segmentation. The overall effect of this correction step is two-fold, such as equalization of intensity fluctuations throughout the image volume, meaning that intensities of identical tissue classes are the same across the image space and increase of inter-tissue contrast, making the statistical separation between different tissue classes (gray matter, white matter, and cerebrospinal fluid) These two effects directly help to improve the segmentation boundary delineation in the following stages of the pipeline.

#### 3.2.2 Isotropic resampling

The voxel spacing is not uniform in different directions when medical images are acquired. Isotropic resampling is used to guarantee similar spatial resolution. Allow the original voxel spacing to be *s*_*x*_, *s*_*y*_, *s*_*z*_. This is expressed as in Equation 3:


$$\begin{array}{l} I_{resampled}\left(i,j,k\right)=\underset{u,v,w}{\sum }I_{corrected}\left(u,v,w\right).\phi_{i}\left(i-u,j-v,k-w\right) \end{array}$$ (3)

where ϕ(.) represents an interpolation kernel, *I*_*corrected*_(*u, v, w*) denotes the bias-corrected intensities at the voxel (*u, v, w*).

#### 3.2.3 Z-score normalization

*Z*-score normalization is used to normalize the intensity values of all images. This is done using Equation 4, where *I*_*resampled*_(*x*) gives the intensity after resampling, μ is the mean intensity and σ is the standard deviation of the image intensity.


$$\begin{array}{l} I_{norm}\left(x\right)=\frac{I_{resampled}\left(x\right)-\mu }{\sigma } \end{array}$$ (4)

**Figure 2:**
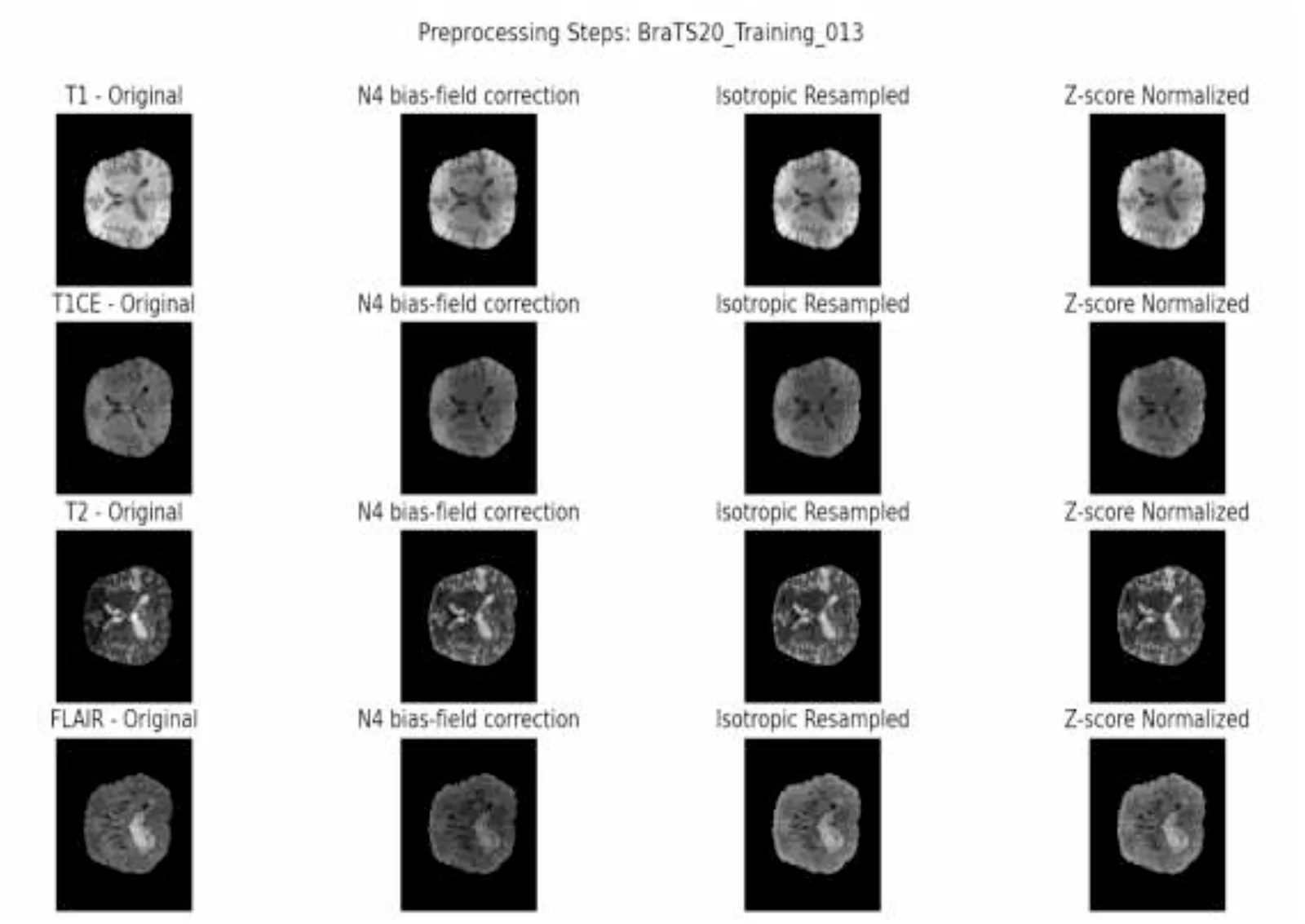
Preprocessed images of the MRI dataset.

**Figure 3:**
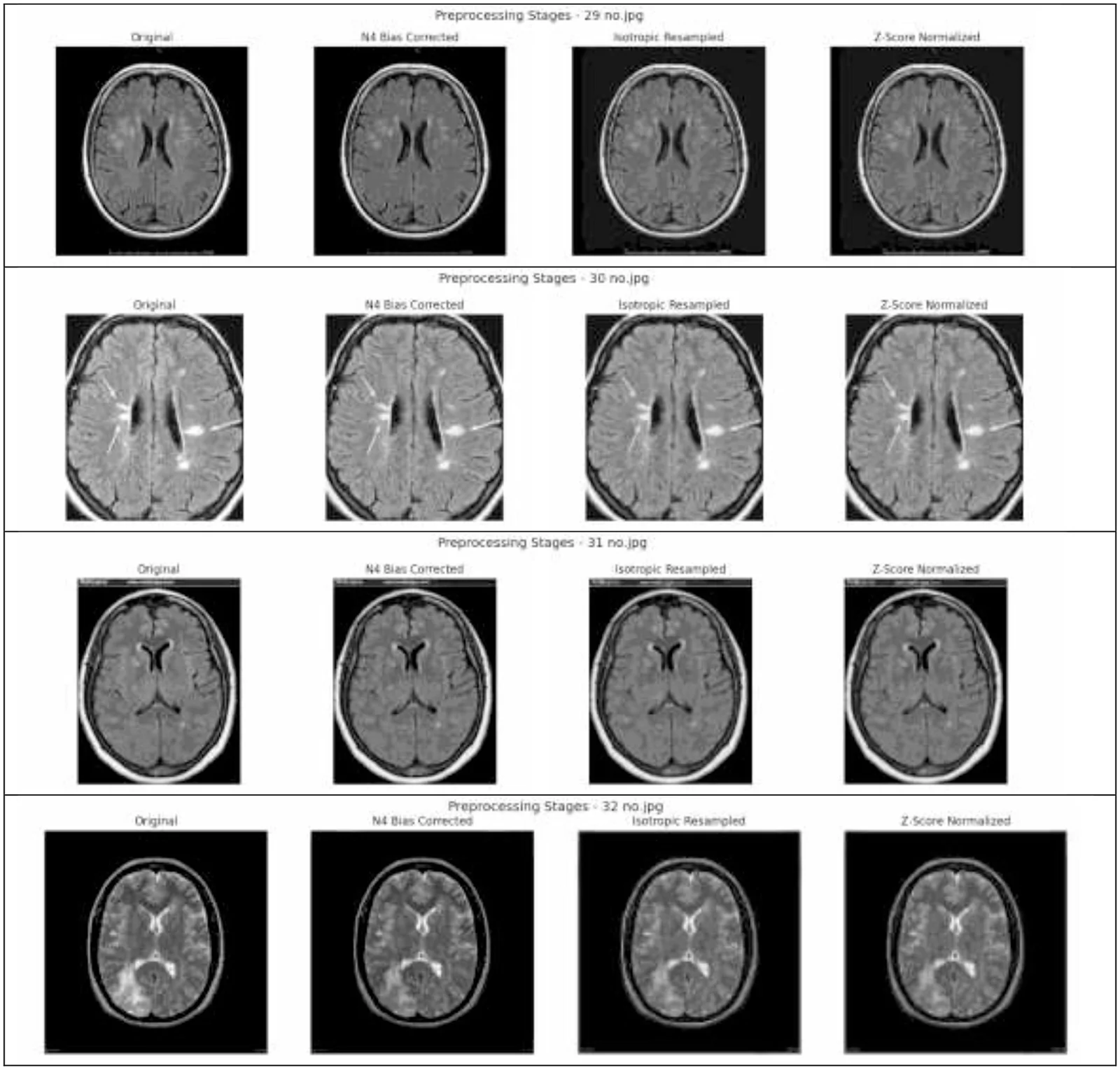
Preprocessed images of the CT dataset.

Such standardization is used to make the image intensities, which enhances the stability and convergence of the following neural network training. Figures 2, 3 show the preprocessed images of the MRI and CT datasets.

#### 3.2.4 Modality-specific augmentations

Modality-specific augmentations are used to make the model more resistant to changes in MRI and CT acquisitions. This is shown in Equation 5, where *A*(.) represents the augmentation operation applied to the image.


$$\begin{array}{l} I_{aug}=A\left(I_{norm}\right) \end{array}$$ (5)

Rotation of MRI images is done to rotate around the three spatial axes, which emulates changes in patient orientation when taking the image. Flipping augmentation takes advantage of the bilateral symmetry of brain structure. The model is trained to identify tumors and tissue borders. Gaussian noise augmentation is the addition of random noise to mimic variability of scanners, and environmental noise, which is sampled according to a normal distribution.

**Figure 4:**
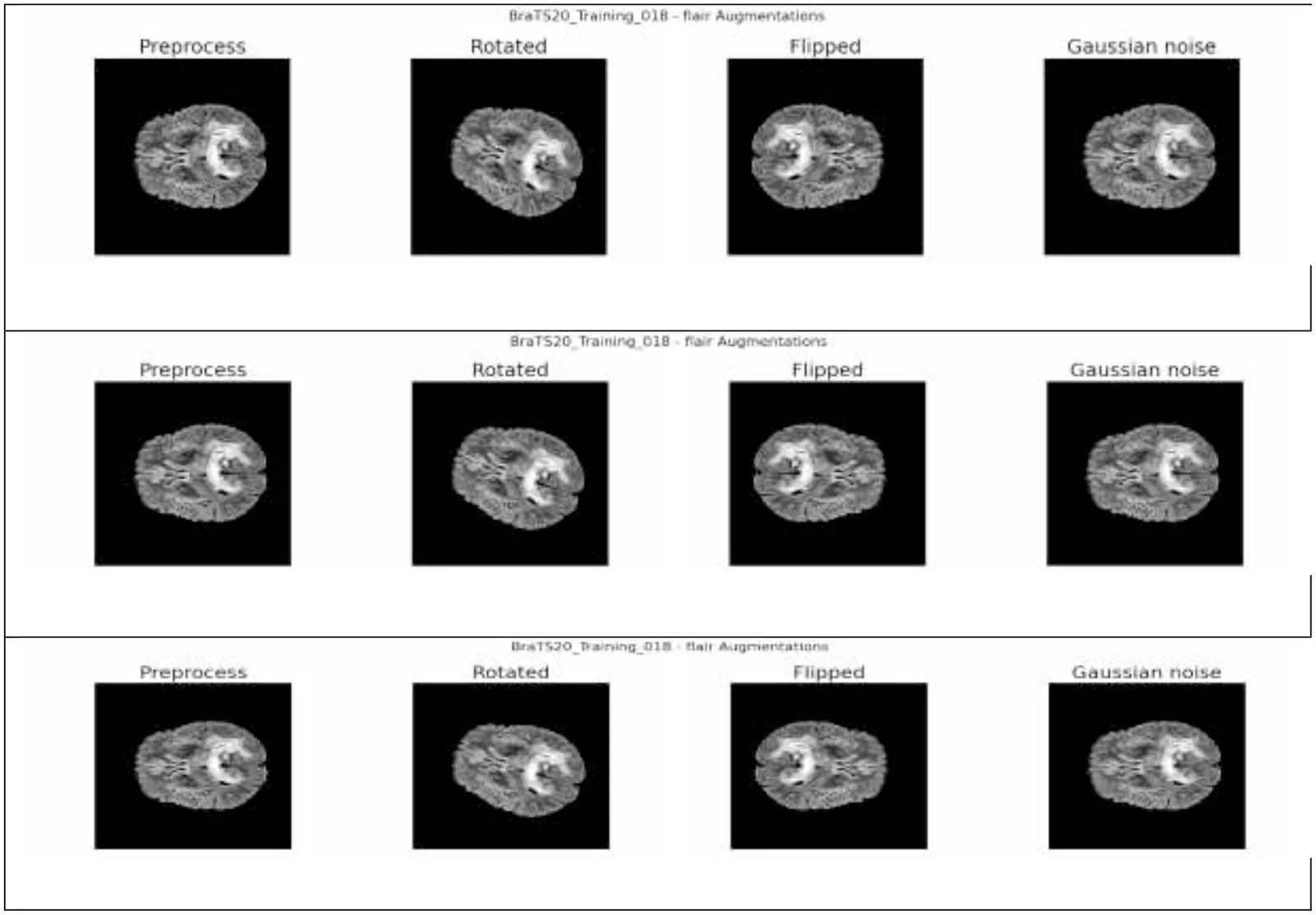
Augmented images of MRI dataset.

**Figure 5:**
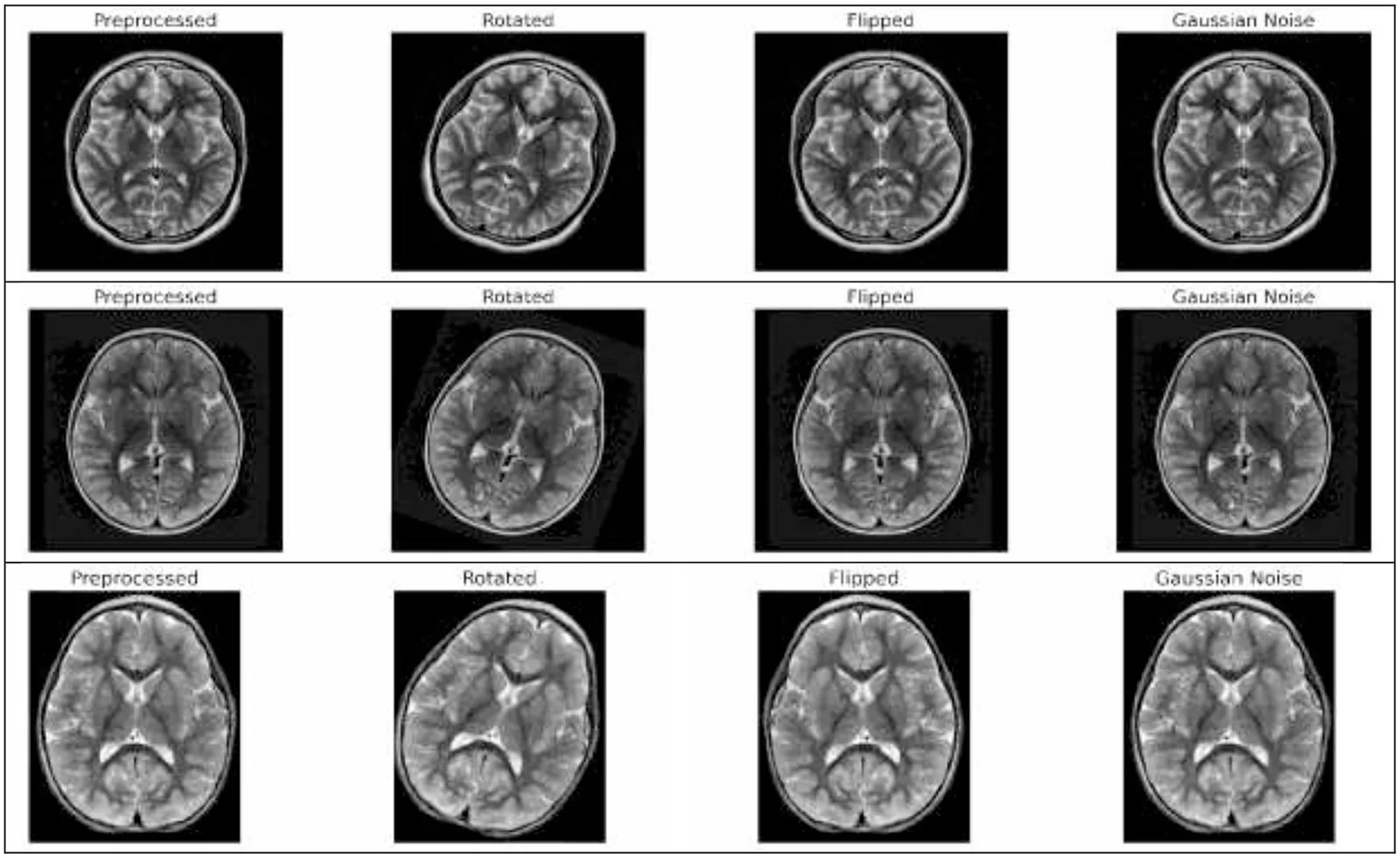
Augmented images of the CT dataset.

Collectively, these augmentations enhance diversity in data sets, decrease overfitting, and improve the accuracy of segmentation in real clinical environments, in which images are in different terms of orientation, symmetry, and noise. Figures 4, 5 show the augmented images of the MRI and CT datasets.

### 3.3 Proposed SUT-Net

**Figure 6:**
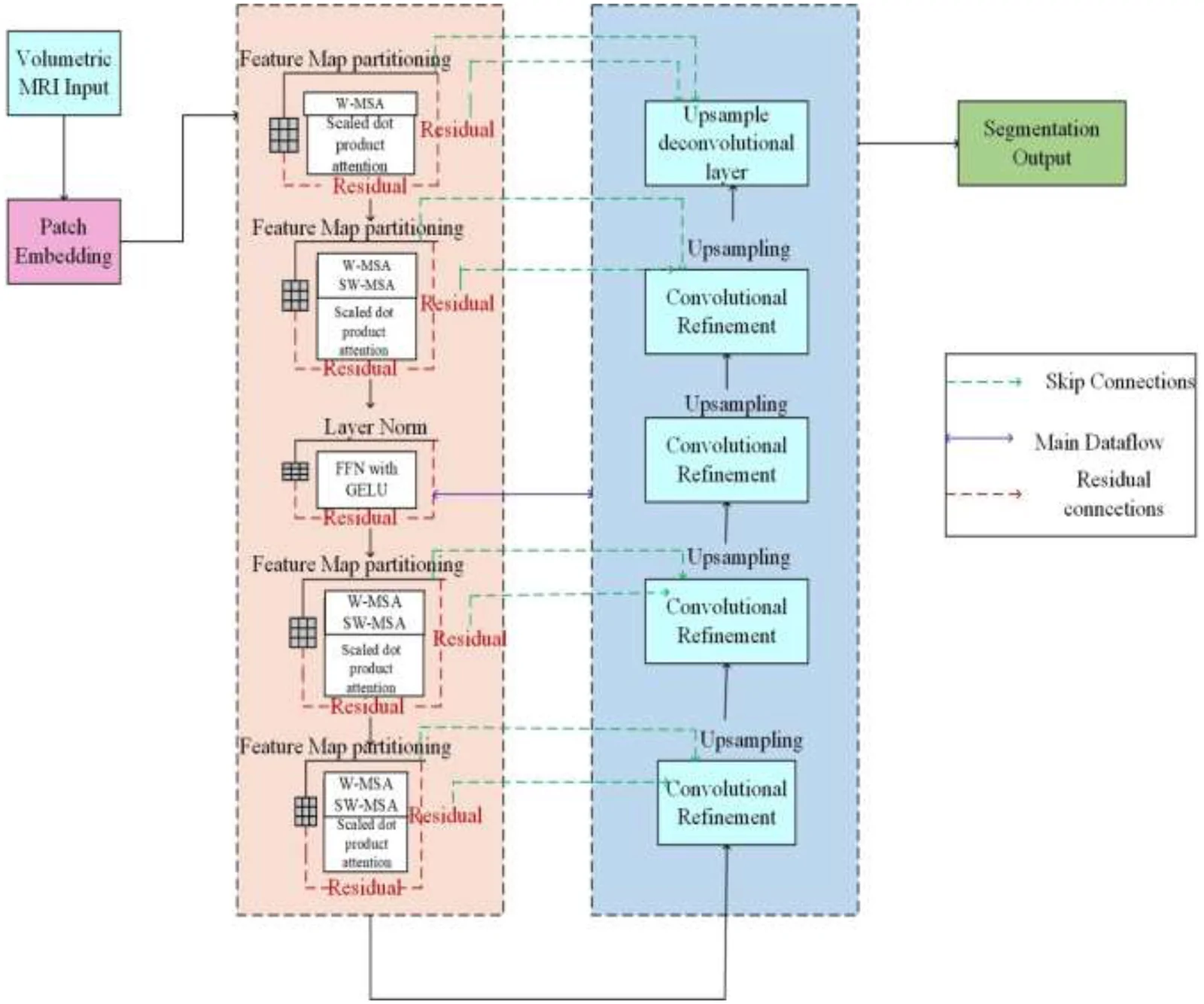
Architecture of the proposed SUT-Net.

The SUT-Net is proposed as a dense segmentation backbone for high-performance brain tumor MRI. It uses hierarchical Swin Transformer (ZongRen et al., 2023) blocks to extract encoder features and a UNETR (Walsh et al., 2022) like decoder to reconstruct high-resolution segmentations. Figure 6 shows the architecture of the proposed SUT-Net. The network is effective in capturing both local structure information and long-range dependencies that are critical to the proper delineation of tumors. The volumetric MRI input is expressed as in Equation 6, when *H, W* denotes height and width of the input image, *D* denotes the depth and *s* denotes the number of channels.


$$\begin{array}{l} X\in R^{H\times W\times D\times C} \end{array}$$ (6)

The encoder derives hierarchical features by successively encoding spatial context with multi-scale transformer blocks. Volumetric MRI data contains high-dimensional spatial data that should be encoded efficiently. To do this, the input is subdivided into non-overlapping 3D patches. This is shown in Equation 7, where, *Patch*(*X*; *P*) shows the Patch partitioning operation with the patch size *P*, *N* denotes the total number of non-overlapping patches and *P*^3^ shows the 3D patch volume.


$$\begin{array}{l} X_{p}=Patch\left(X;P\right)\;X_{p}\in R^{N\times P^{3}C} \end{array}$$ (7)

The patches are then mapped to a fixed embedding dimension. This is expressed using Equation 8, where, *Z*_0_ denotes initial transformer input embedding, *X*_*p*_ is the patch embeddings, *E* represents the learnable linear projection matrix and *E*_*pos*_ is a positional embedding vector.


$$\begin{array}{l} Z_{0}=X_{p}E+E_{pos} \end{array}$$ (8)

Transformer blocks are each created to learn to refine the encoded characteristics by modeling of contextual relationships between patches of the image, as shown using Equation 9, where ${\tilde{Z}}^{\left(l\right)}$ denotes normalized feature, *LN*(·) is the layer normalization and *Z*^(*l*)^ is the input feature representation of the layer *l*.


$$\begin{array}{l} {\tilde{Z}}^{\left(l\right)}=LN(Z^{\left(l\right)} \end{array}$$ (9)

The normalized features are then projected to separate subspaces to create query, key and value representations. After creating queries, keys, and values, the model then calculates attention scores to decide how much attention is to be given to various patches compared to others. Scaled dot-product attention is used as shown in Equation 10 where *dk* represents a key dimension, *B* denotes the attention bias.


$$\begin{array}{l} Attn\left(Q,K,V\right)=Softmax\left(\frac{QK^{T}}{\sqrt{dk}}+B\right)V \end{array}$$ (10)

To further improve feature extraction, the attention is spread over a series of heads. The heads are complementary in their dependencies, and their products are more enriched representations. This is called multi-head self-attention and represented as in Equation 11 where, *h* denotes the number of attention heads.


$$\begin{array}{l} MSA\left({\tilde{Z}}^{\left(l\right)}\right)=Concat\left[head_{1},\ldots ,head_{h}\right]W_{o} \end{array}$$ (11)

The connections are then added back in to stabilize optimization and allow a smoother gradient flow to prevent loss of information by the deep layers. The new feature representation post-attention is written as in Equation 12, *U*^(*l*)^ denotes the post-attention residual block output.


$$\begin{array}{l} U^{\left(l\right)}=Z^{\left(l\right)}+MSA\left({\tilde{Z}}^{\left(l\right)}\right) \end{array}$$ (12)

Another normalization step is used after the addition of the residuals to enhance stability. This is the normalized version, which is defined as in Equation 13, where Ũ^(*l*)^is normalized post-attention feature.


$$\begin{array}{l} {Ũ}^{\left(l\right)}=LN\left(U^{\left(l\right)}\right) \end{array}$$ (13)

After the attention and normalization, a two-layer feed-forward network (MLP) adds non-linearity to the model. To improve the features abstraction with smooth gradient properties, the GELU activation function is applied, which is shown in Equation 14.


$$\begin{array}{l} MLP\left({Ũ}^{\left(l\right)}\right)=\sigma \left(U^{\left(l\right)}W_{1}+b_{1}\right)W_{2}+b_{2} \end{array}$$ (14)

Lastly, one more residual linkage combines the MLP output and the existing representation to generate the refined output of the transformer block. This is formulated as in Equation 15.


$$\begin{array}{l} Z^{\left(l+1\right)}=U^{\left(l\right)}+MLP\left({Ũ}^{\left(l\right)}\right) \end{array}$$ (15)

The model is stacked with *L* transformer blocks to hierarchically represent both fine-grained and global structures, extracting fine-grained features in brain MRI images accurately.

To be able to model dependencies between local regions without paying too high cost in computation, the feature map is broken into fixed-size windows. This is expressed using Equation 16, where *M* is the size of the window and *W*_*i*_ denotes the tokens.


$$\begin{array}{l} W_{i}=WindowPartition\left(Z^{\left(l\right)};M\right) \end{array}$$ (16)

The window positions are offset by a distance Δ to further increase the size of the receptive field and allow cross-window relationships to be captured in alternate layers. This is shown as in Equation 17, where is the shift offset.


$$\begin{array}{l} W_{i}^{shift}=CyclicShift\left(W_{i};\Delta \right) \end{array}$$ (17)

Attention is then calculated separately in each window after being shifted. The model uses scaled dot-product attention at the window level to selectively highlight the most relevant local features with computational tractability. This mechanism is written as in Equation 18.


$$\begin{array}{l} SW-Attn\left(W_{i}^{shift}\right)=Softmax\left(\frac{QK^{T}}{\sqrt{dk}}+B_{sw}\right)V \end{array}$$ (18)

Lastly, the results of each window are combined and inverted-shifted to form the entire feature map. This ensures worldwide uniformity and still has the advantage of local focus. This is represented in Equation 19, where *SW* − *Attn*(·) denotes a shifted window attention operation, *Q, K, V* denotes the query, key and value matrix, *d*_*k*_ denotes the dimension of key vectors and *B*_*sw*_ is shifted-window attention bias.


$$\begin{array}{l} Z_{merged\left(l\right)}=ReverseShift\left(MergeWindows\left(\left\{SW-Attn\right\}\right)\right) \end{array}$$ (19)

The encoder down-samples the spatial resolution but upscales the feature dimensionality in a progressive way to learn multi-scale representations. This is achieved by patch merging, in which adjacent patch embeddings are merged and linearly projected into a higher-dimensional space. This is formulated as using Equation 20, where *Z*^(*s*)^ is the token at the encoder stage.


$$\begin{array}{l} Z^{\left(S+1\right)}=PatchMerge\left(Z^{\left(s\right)}\right) \end{array}$$ (20)

The down-sampling is followed by a series of Swin stages, each processing all the resolution levels using several layers of shifted-window attention and feed-forward networks are expressed using Equation 21, where *F*^(*s*)^ is the output feature map.


$$\begin{array}{l} F^{\left(s\right)}=SwinStage\left(Z^{\left(s\right)}\right),s=0,1,\ldots ,S-1 \end{array}$$ (21)

The framework provides a balance between the local and global structural awareness that is essential to the segmentation of brain tumors through this multi-resolution encoding.

The decoder is used to reconstruct high-resolution segmentation maps by progressively up-sampling the encoder features with skip connections. Encoder characteristics are reformatted to the decoder resolution to allow skip connections to be spatially aligned. These are shown in Equation 22.


$$\begin{array}{l} F_{vol}^{\left(s\right)}=Unflatten\left(F^{\left(s\right)}\right) \end{array}$$ (22)

The decoder up-samples the last feature map recursively and combines it with the encoder features to preserve fine spatial information. This is expressed in Equation 23, where *D*^(*i*)^ denotes the decoder feature, *D*^(*i*−1)^ shows the previous decoder feature.


$$\begin{array}{l} D^{\left(i\right)}=UpSample\left(D^{\left(i-1\right)}\right)⊕F^{\left(s=1\right)} \end{array}$$ (23)

Once concatenated, a convolutional block is used to refine the features to make tumor edges clear and artifacts minimal, which is expressed using Equation 24


$$\begin{array}{l} D^{\left(i\right)}\leftarrow ConvBlock\left(D^{\left(i\right)}\right) \end{array}$$ (24)

**Figure 7:**
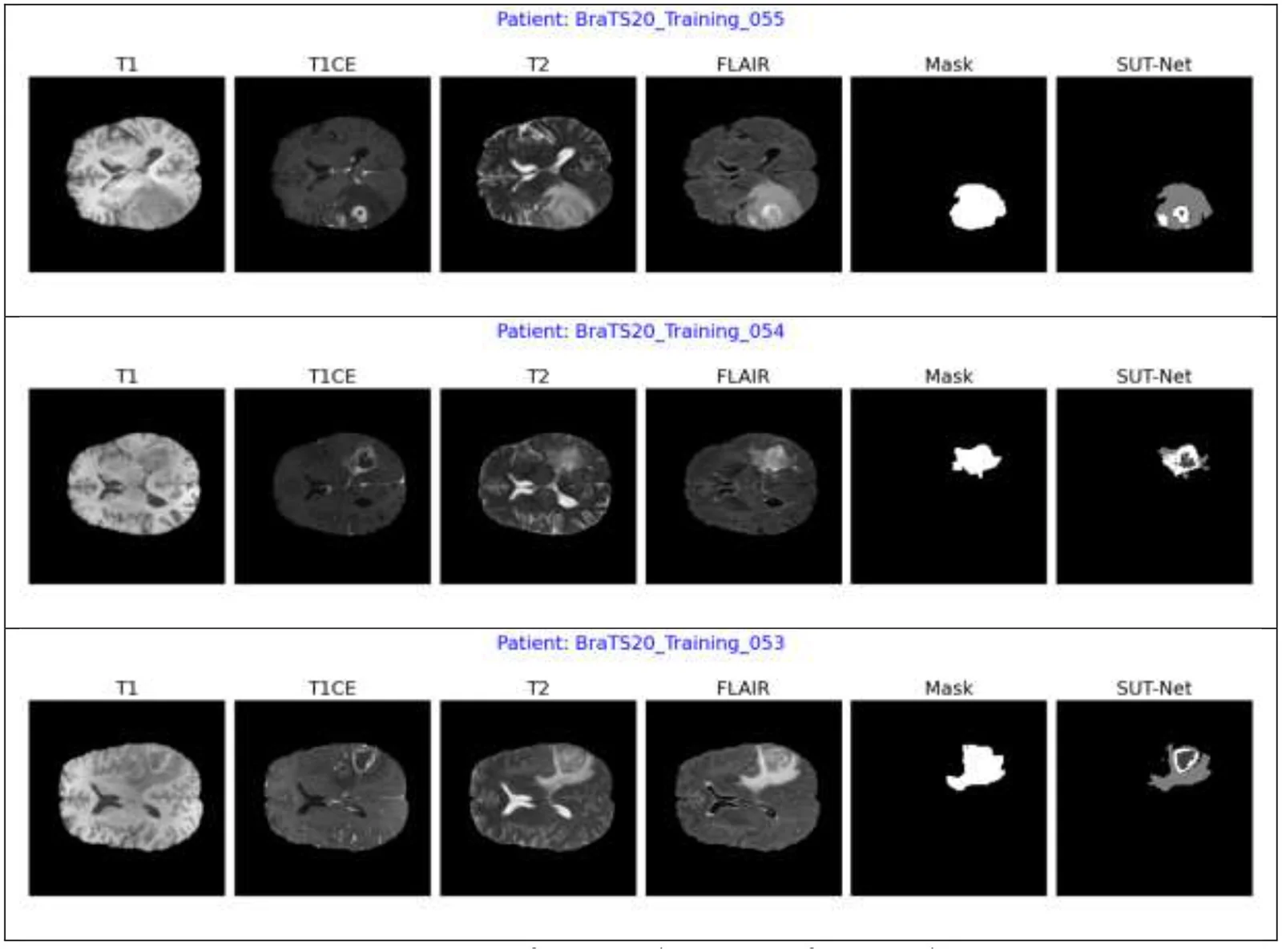
Output of proposed SUT-Net for MRI dataset.

**Figure 8:**
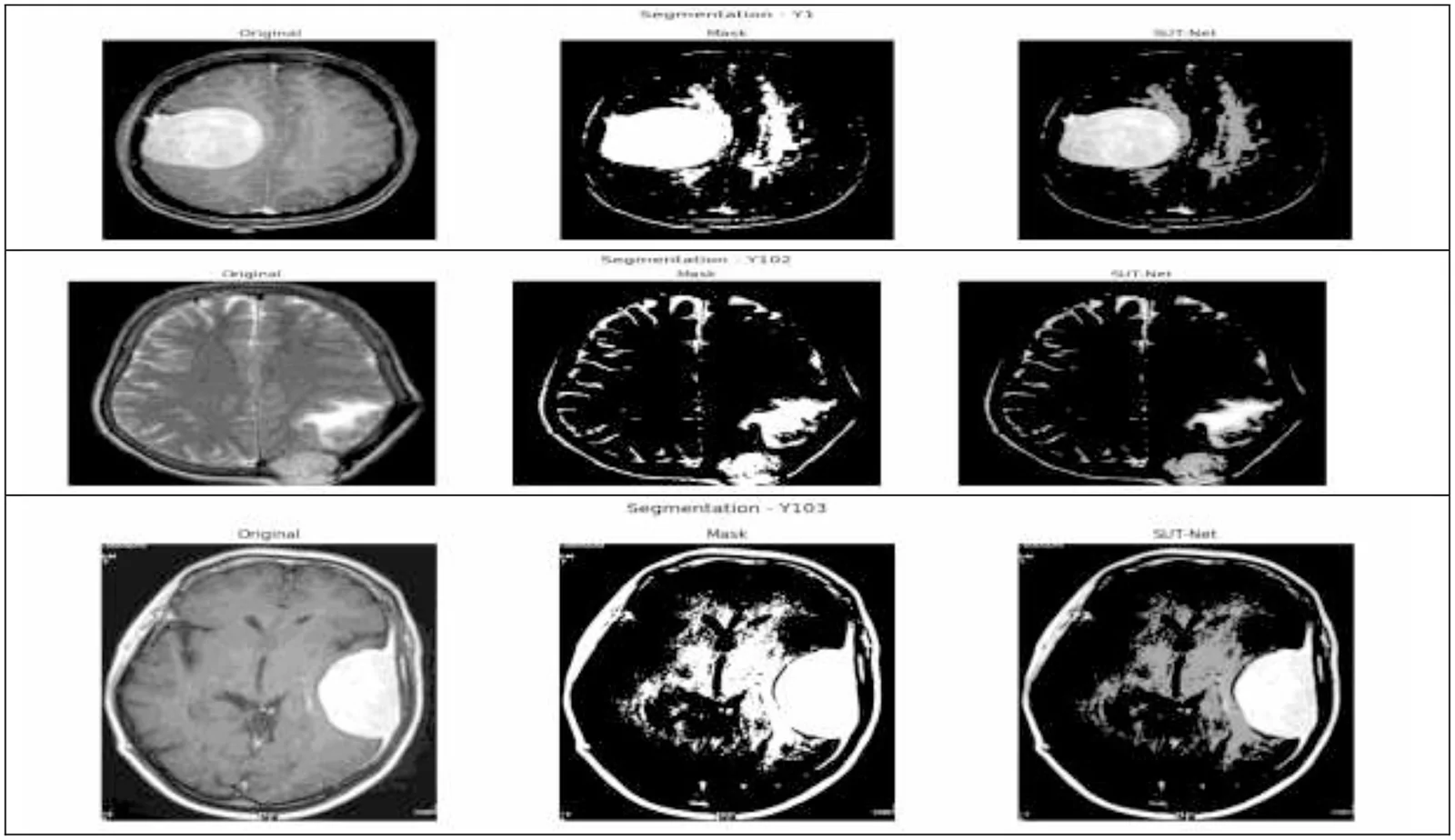
Output of proposed SUT-Net for CT dataset.

This architecture enables SUT-Net to effectively model fine-grained tumor morphology and long-range spatial relationships, thus effectively segmenting multi-modal brain MRI. Figures 7, 8 show the output of the proposed SUT-Net for MRI and CT datasets.

### 3.4 Neuromorphic encoding and refinement

**Figure 9:**
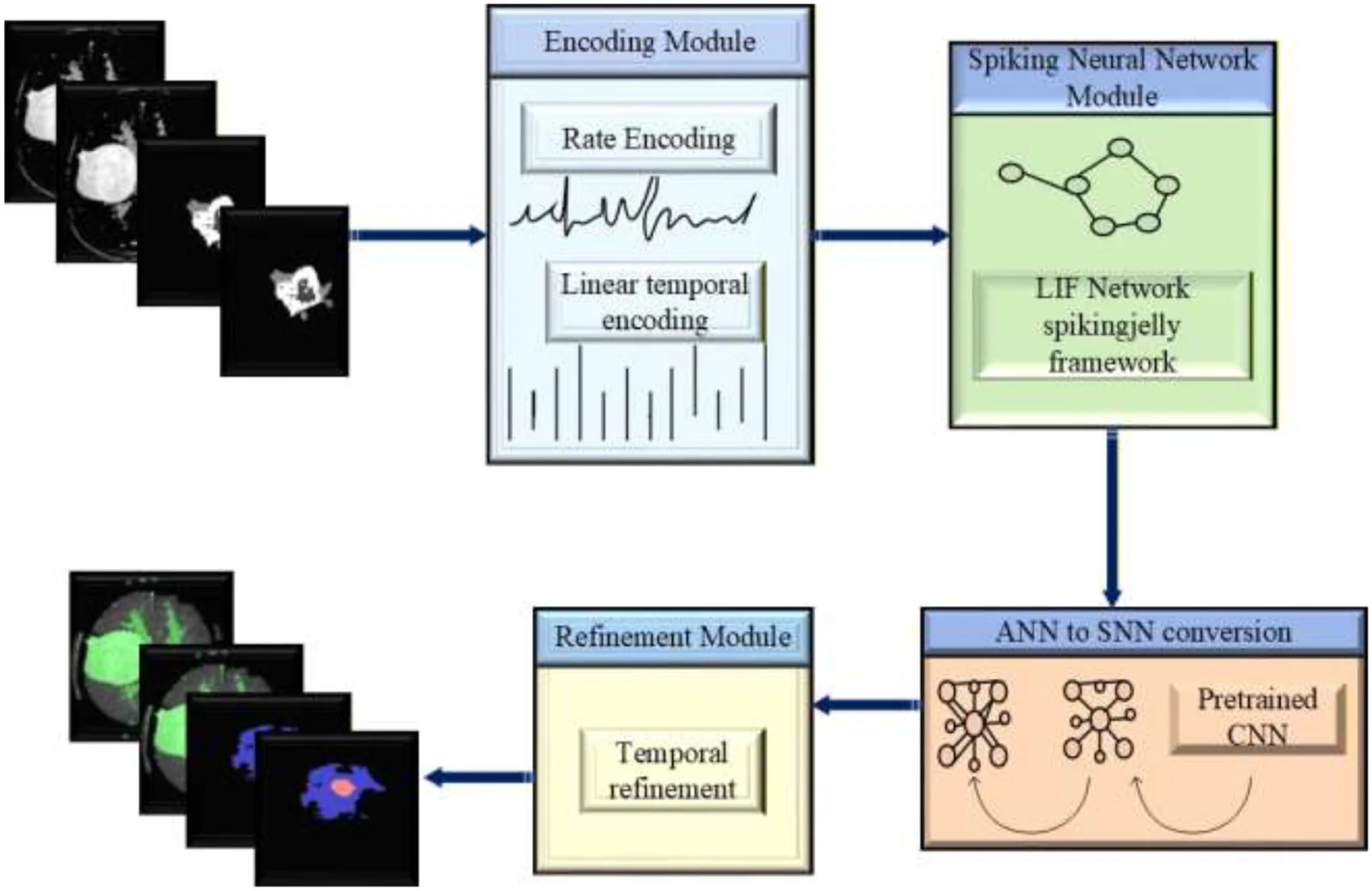
Neuromorphic encoding and refinement.

SPiKe-Med introduces neuromorphic-inspired processing to provide refinement of segmentation with energy-efficient and accurate results. SUT-Net backbone multi-scale feature maps are transformed into spike trains and fed into a LIF (Lu and Xu, 2022) with SpikingJelly (Fang et al., 2023). It simulates the biological neural dynamics and saves computational cost without compromising the segmentation performance. SPiKe-Med utilizes both the temporal spike encoding and the transformer-based attention for learning spatial and temporal context. Swin Transformer learns long-range spatial relationships using global self-attention and spike-based temporal encoding, keeping the activation of a feature for more than one time step by using the LIF dynamics. Such accumulation allows the network to keep necessary information and filter noise, which in turn produces better segmentation for the jagged tumor border. Sparse spike-based computation also leads to better energy efficiency performance without affecting the segmentation accuracy. Figure 9 shows the Neuromorphic Encoding and Refinement process.

#### 3.4.1 Temporal encoding of features into spike trains

Multi-scale features are encoded into temporal spike trains to interface them with a spiking network. One of them is rate-based temporal encoding, in which the strength of each feature is used to compute the likelihood of spike production at discrete time steps *T*. Let *F*(*v*) is the value of the feature at the voxel *v*, the spike train at voxel *s*(*x, t*) defined as in Equation 25, where θ_*t*_ denotes the probability threshold.


$$\begin{array}{l} S\left(v,t\right)=\{\begin{array}{c} 1,\;if\;F\left(v\right)>\theta_{t}\\ 0,\;otherwise \end{array} \end{array}$$ (25)

This makes sure that more high-intensity feature activations produce spikes at multiple time steps, and fewer or no spikes are produced by low-intensity features, resulting in sparsity. Alternatively, temporal encoding using linear scaling is applied. This is expressed in Equation 26, where *F*_max_ is the maximum feature value in the map, *F*_min_ is the minimum feature value in the map. Here, the voxels, which have stronger activations, result in earlier spikes, whereas the weaker activations are postponed, thus encoding the information into the temporal domain. Early spikes highlight important anatomical features, so that the SNN focuses on important regions in the refinement phase.


$$\begin{array}{l} t_{spike}\left(x\right)=T\left(1-\frac{F\left(x\right)-F_{\min}}{F_{\max}-F_{\min}}\right) \end{array}$$ (26)

This is an efficient temporal encoding system that fills the divide between fixed feature maps and event-based neuromorphic computation, adding temporal variation to the representation of medical images.

#### 3.4.2 Refinement with LIF-based spiking neural network

The spike trains are input into a LIF-based SNN (Duan et al., 2025), which is a model of the membrane potential dynamics of biological neurons. The membrane potential of a neuron *i*(*t*) is changing in a manner that was shown in Equations 27, 28, where *V*_*i*_(*t*) denotes membrane potential, *I*_*i*_(*t*) input current in the neuron.


$$\begin{array}{l} \tau_{m}\frac{dV_{i}\left(t\right)}{dt}=-V_{i}\left(t\right)+I_{i}\left(t\right) \end{array}$$ (27)

$$\begin{array}{lll} V_{i}\left(t\right) & \to & V_{reset} \end{array}$$ (28)

This LIF model enables the network to store evidence as time goes on, and only fires when enough input is obtained, thus making sparse computation energy-efficient. The network further refines the original segmentation with a combination of multi-scale feature representation and temporal dynamics. The training plan is aimed at providing correct segmentation, constant convergence, and energy-efficient inference.

Spiking neurons produce non-differentiable binary spike outputs, which prevents the direct use of standard backpropagation. Surrogate gradients address this limitation by approximating the spike derivative with a smooth function, enabling stable gradient-based learning. They were adopted in this research to support end-to-end training of SNN while preserving temporal dynamics and ensuring efficient convergence. A neuron with a membrane potential is said to have a potential of *V* and threshold *V*_*th*_, the surrogate gradient is given as in Equation 29, where *S* is the spike output, σ′ denotes the differentiable function.


$$\begin{array}{l} \frac{\delta S}{\delta V}\approx \sigma^{\prime }\left(V-V_{th}\right) \end{array}$$ (29)

Standard backpropagation is used to train the LIF-based SNN layers using surrogate gradients, and thus the network is trained to learn both temporal and spatial patterns. To use the weights of pretrained CNN (Rao et al., 2024) encoders, a partial ANN-to-SNN conversion is used. The FCNN continuous-valued feature activations of the pretrained CNN are rate-coded into the spiking domain. This is shown using Equation 30.


$$\begin{array}{l} S_{i}\left(t\right)=\{\begin{array}{c} 1,\;if\;F_{CNN,i}>\theta \\ 0,\;otherwise \end{array} \end{array}$$ (30)

The methodology saves training time, enhances convergence, preserves pretrained feature extraction functionality and makes ultra-low latency inference in the spiking domain possible. It has been demonstrated that to achieve accurate segmentation in medical imaging, particularly of brain tumors, a variety of spatial overlap accuracy measures are combined with robust class imbalance and temporal consistency in spiking networks. To achieve this, SPiKe-Med employs a composite multi-objective loss function, which combines Dice loss, Focal loss, and Temporal consistency loss. This is expressed in Equation 31.


$$\begin{array}{l} L_{total}=\lambda_{1}L_{Dice}+\lambda_{2}L_{Focal}+\lambda_{3}L_{Temporal} \end{array}$$ (31)

Dice loss measures the coincidence of the predicted segmentation Ŷ and ground truth *Y*, which is the direct optimization of the segmentation performance of voxel-wise predictions. It is defined as in Equation 32. Dice loss is also very useful when dealing with unbalanced tumor regions, in which small structures are underrepresented.


$$\begin{array}{l} L_{Dice}=1-\frac{2\sum_{i}{Ŷ}_{i}Y_{i}}{\sum_{i}{Ŷ}_{i}+\sum_{i}Y_{i}} \end{array}$$ (32)

Focal loss is used to further reduce the problem of class imbalance as well as to concentrate on voxels that are difficult to classify. This is defined in Equation 33


$$\begin{array}{l} L_{Focal}=-\underset{i}{\sum }\alpha_{i}{\left(1-{Ŷ}_{i}\right)}^{\gamma }Y_{i}\log\left({Ŷ}_{i}\right) \end{array}$$ (33)

Spike trains are time-coded; the network should be able to sustain constant spiking patterns throughout time. Temporal consistency loss is a regularity loss that discourages sudden spike activity changes between successive time steps *t*. This promotes spiking dynamics that are smooth, so that the SNN produces consistent and predictable predictions, but at the same time, the SNN is coherent in time. This is expressed in Equation 34.


$$\begin{array}{l} L_{Temporal}=\frac{1}{T}\overset{T}{\underset{t=1}{\sum }}\|S\left(t\right)-S\left(t-1\right)\|\begin{array}{c} 2\\ 2 \end{array} \end{array}$$ (34)

These three losses put together guarantee that SPiKe-Med is able to optimize spatial accuracy, class balance and temporal stability simultaneously, which is essential in clinical use. Neural networks give overconfident or underconfident probability estimates even after training. Clinical decision-making in medical imaging requires well-calibrated predictions. As a post-processing procedure, SPiKe-Med uses temperature scaling to scale the confidence of predicted probabilities. This is shown in Equation 35, where ${Ŷ}_{i}^{cal}$ denotes the calibrated probability, *z*_*i*_ represent the logits.


$$\begin{array}{l} {Ŷ}_{i}^{cal}=Softmax\left(\frac{z_{i}}{\tau }\right) \end{array}$$ (35)

**Figure 10:**
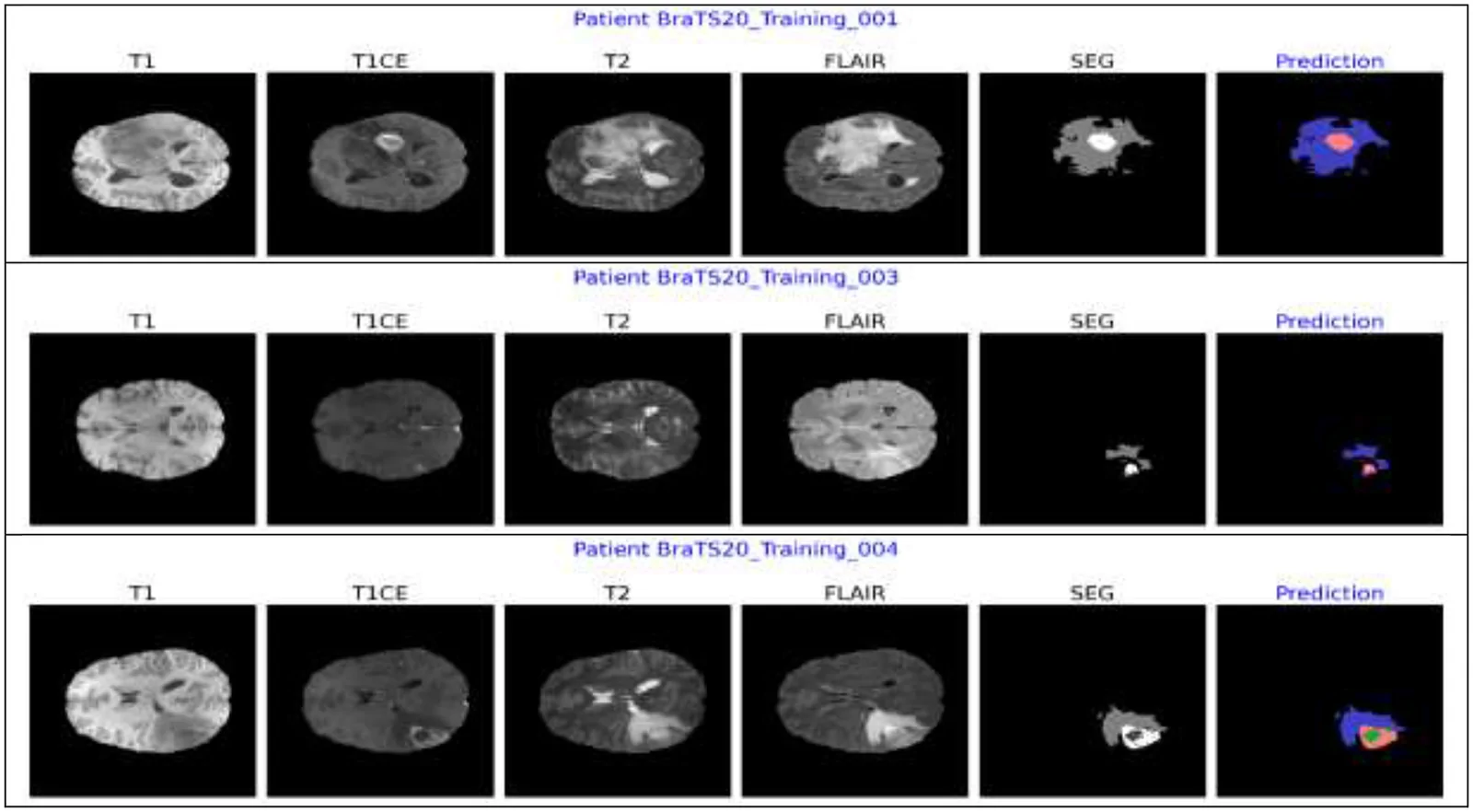
Final segmentation output for the MRI dataset.

**Figure 11:**
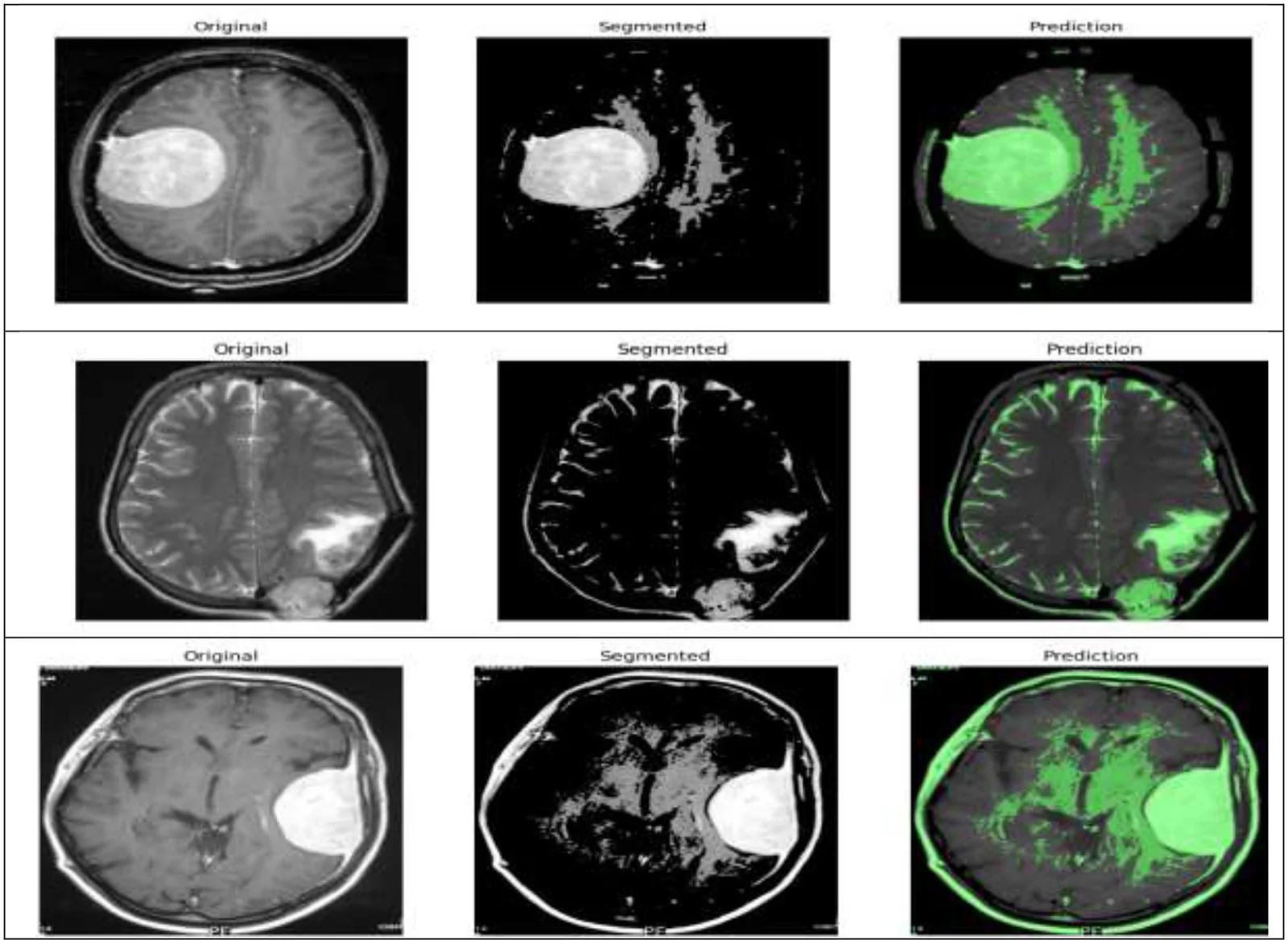
Final segmentation output for the CT dataset.

Temperature scaling has no impact on prediction ranking but enhances reliability and calibration, especially when the prediction is voxel-wise in the case of clinical MRI and CT scans. SPiKe-Med is able to provide accurate and reliable segmentation results by using multi-objective loss optimization and temperature scaling. Figures 10, 11 show the final segmentation output of the MRI and CT datasets.

Table 2 presents a summary of the main training and optimization parameters to pretrain the ANN, fine-tune, and SNN surrogate-gradient training in the SPiKe-Med framework. These environments were chosen to have steady convergence, equal loss minimization and equal comparison of all the models that were evaluated.

**Table 2:** Describes the training and optimization parameters.

Training parameter	Configuration
Learning rate (ANN)	1e-3
Learning rate (ANN)	1e-4
Learning rate (SNN training)	1e-4 → 5e-6 (cosine decay)
Optimizer	AdamW
Weight decay	1e-5
Gradient clipping	1.0
Dice loss weight	0.5
Focal loss weight	0.3
Temporal loss weight	0.2
Focal loss γ	2.0
Focal loss α	0.25
Temperature scaling (T)	2.0 → 0.5
ANN pre-training epochs	50–80
ANN fine-tuning epochs	20–30
SNN surrogate-gradient epochs	100–150
Final SNN stabilization epochs	20–30
Batch size (ANN)	16–32
Batch size (SNN)	4–8
Inference batch size	1

Table 3 shows the main hyperparameters of the LIF neuron model of the neuromorphic refinement part of the proposed framework. The parameter choice had to ensure the stability of spike propagation in time, efficient feature refinement and energy saving while analyzing brain tumor images.

**Table 3:** Configuration parameters of LIF neurons in the proposed SPiKe-Med framework.

Parameter	Value
Membrane time constant (τ)	20 ms
Firing threshold (Vth)	1.0
Reset potential (Vreset)	0
Simulation time steps (T)	8–10
Surrogate gradient	Sigmoid surrogate
Spike encoding	Rate-based temporal encoding
Membrane leak factor	0.95

## 4 Results and discussions

The SPiKe-Med framework is tested using a multimodal MRI dataset consisting of four modalities (T1, T1ce, T2, and FLAIR) [https://www.kaggle.com/datasets/awsaf49/brats20-dataset-training-validation/data]. A CT brain dataset of 2D images [https://www.kaggle.com/datasets/drskprabhakar/brain-tumor-ct-images-binary-classification]. All experiments were performed on a computer with an Intel Core i5 (10 th Gen), 16 GB of RAM, and an NVIDIA GTX 1,650 graphics card with 4 GB of VRAM. The software platform was based on a 64-bit Windows 11 operating system and Python 3.11, and was developed using the PyCharm integrated development environment. The dataset was split into training and testing sets using a 70:30 ratio. Model performance was evaluated on the respective test sets. These models provide a reasonable comparative level, upon which the performance of the proposed SPiKe-Med framework is evaluated. Gatransformer (Tehsin et al., 2025), ViT-CB (Tanone et al., 2025), ConvNeXt (Lopez-Ramirez et al., 2026), and BrainDx (Kaur et al., 2026) were chosen as the most common brain tumor segmentation models that represent recent DL methods, which allows making a fair comparison with the suggested SPiKe-Med framework.

### 4.1 Implementation details and experimental protocol

The complete implementation workflow of the proposed SPiKe-Med framework is illustrated through the following experiments. Firstly, both MRI and CT images were processed using the preprocessing pipeline that includes N4 bias field correction, isotropic resampling, *z*-score normalization, image resizing, and data augmentation for each imaging modality. It should be noted that the data augmentation procedure was performed only on the training dataset, while the validation datasets were not augmented for unbiased evaluation.

The preprocessed datasets were divided into training and testing subsets using a 70:30 ratio. The training subset was used for model training, while the testing subset was reserved exclusively for performance evaluation. First, the SUT-Net backbone was trained for spatial features extraction, and then the obtained features were encoded in temporal spike trains using the temporal encoding algorithm proposed in this paper. After that, the refinement of the spike representations was performed using the LIF spiking neural network optimized via the surrogate gradient algorithm. The composite loss function, including Dice loss, Focal loss, and Temporal Consistency loss, was used during training.

The hyperparameters were empirically tuned by utilizing the validation dataset. Various learning rates, batch sizes, and optimization schemes were tried, and the best configuration shown in Tables 2, 3 was chosen due to its stable convergence and better performance in the validation phase. AdamW optimizer, learning rate decay, and gradient clipping were used in the optimization procedure to enhance the training process. The network that yielded the maximum validation Dice score was considered the final network to be tested. All the models used in comparisons were trained and evaluated under the same conditions. The reported energy per inference values were estimated using the computational cost of the models rather than direct hardware power measurements.

Energy consumption was calculated from the inference latency and computational complexity (GFLOPs), providing a consistent software-based estimation using the thop profiling library for comparing different models under identical hardware and experimental conditions. The energy per inference formula estimates the total energy consumed by a neural network to process one input sample. It is calculated by adding the energy used by all layers in the network. This is given in Equation 36. For each layer, the number of computational operations (*N*_*l*_) is multiplied by the energy required for a single operation (*E*_*op*_). The sum of these values across all *L* layers gives the total inference energy (*E*_inf_), which is usually expressed in microjoules (μJ). This metric is useful for evaluating the energy efficiency of deep learning models, especially when deploying them on edge devices or embedded systems with limited power resources.


$$\begin{array}{l} E_{\inf}=\overset{L}{\underset{l=1}{\sum }}N_{l}\cdot E_{op} \end{array}$$ (36)

### 4.2 Performance metrices

The performance metrics are used to assess the effectiveness of a model. They evaluate different properties, including the accuracy of segmentation, boundary accuracy, computational efficiency, and overall prediction accuracy. These measures are used to compare the various models and make sure that the model works consistently on various datasets and tasks. Table 4 shows the metrices used to evaluate the performance of the proposed model.

**Table 4:** Summary of performance metrices used to evaluate the proposed model.

Metrices	Formula	Definition
Dice score %	$Dice\;Score=\frac{2\cdot |P\cap G|}{|P|+|G|}$	Measures the intersection-to-union ratio
IOU %	$IoU=\frac{|P\cap G|}{|P\cup G|}$	It is a metric of the nearest distance between the prediction and ground truth boundary points
Hausdorff distance (mm)	*HD*(*P, G*)=$\max\left\{\begin{array}{c} \sup\\ p\in P \end{array}\begin{array}{c} \inf\\ g\in G \end{array}\;d\left(p,g\right),\begin{array}{c} \sup\\ g\in G \end{array}\begin{array}{c} \inf\\ p\in P \end{array}\;d\left(g,p\right)\right\}$	The distance between prediction and ground truth boundary points is measured
Spike-rate (Hz)	$Spike-rate=\frac{Total\;spikes\;fired}{Total\;possible\;spikes}$	Share of active spikes of a spiking neural network during inference
Binary cross-entropy (BCE) loss	$BCE\;loss=-\frac{1}{N}\overset{N}{\underset{i=1}{\sum }}y_{i}\log\left({ŷ}_{i}\right)+\left(1-y_{i}\right)log\left(1-{ŷ}_{i}\right)$	Applied in pixel-wise classification in segmentation
Classification accuracy %	$Accuracy=\frac{Number\;of\;correct\;predictions}{Total\;number\;of\;predictions}$	Percentage of correct classification of pixels
Energy per inference (microjoules)	$E_{\inf}=\overset{L}{\underset{l=1}{\sum }}N_{l}\cdot E_{op}$	Total energy consumed during one forward pass
Accuracy %	$Accuracy=\frac{TP+TN}{TP+FP+FN}$	Fraction of correctly identified instances of total instances
Precision %	$Precision=\frac{TP}{TP+EP}$	Proportion of correctly predicted positives
Sensitivity %	$Sensitivity=\frac{TP}{TP+FN}$	Fraction of the true positives identified
Specificity %	$Specificity=\frac{TN}{TN+FP}$	Fraction of true negatives identified
F-measure %	$F1+\frac{2\times Precision\times Recall}{Precision+Recall}=\frac{2TP}{2TP+FP+FN}$	Harmonic mean of Precision and Recall
MCC	$MCC=\frac{TP\times TN-FP\times FN}{\sqrt{\left(TP+FP\right)\left(TP+FN\right)\left(TN+FP\right)\left(TN+FN\right)}}$	Correlation coefficient of predicted and observed classes
MSE	$MSE=\frac{1}{N}\overset{N}{\underset{i=1}{\sum }}{\left(yi-\hat{y\;}i\right)}^{2}$	Mean squared error in predicted probabilities and observed class values
NPV %	$NPV=\frac{TN}{TN+FN}$	The fraction of negatives that are correctly predicted of all predicted negatives
FNR %	$FNR=\frac{FN}{TP+FN}$	Ratio of the real positives that are falsely classified as negatives
FPR %	$FPR=\frac{FP}{FP+TN}$	Proportion of negatives incorrectly classified as positives
GMean	$\sqrt{Sensitivity\cdot Specificity}$	Measures the balance between sensitivity and specificity
Cohen's kappa	$\kappa =\frac{p_{o}-p_{e}}{1-p_{e}}$	Measures the level of agreement between predicted and actual labels

### 4.3 Comparative analysis

The baseline models were selected because they are popular models used in brain tumor segmentation, and they reflect both the traditional and new DL models. These models represent the state-of-the-practice and allow for making a fair comparison with the proposed SPiKe-Med framework.

Performance comparisons between the SPiKe-Med architecture and other algorithms on the MRI dataset have been shown in Table 5. All of the reported metrics have been calculated as mean ± standard deviation based on multiple experimental runs. The SPiKe-Med model recorded the best Dice Score (0.9728 ± 0.010) and IoU Score (0.9584 ± 0.011), and the least Energy per Inference (0.41 ± 0.021 μJ) amongst all of the compared models. Moreover, the SPiKe-Med model performed better than all of the competing models in terms of segmentation and classification metrics.

**Table 5:** Comparative performance of existing models on the MRI dataset.

Metric	Gatransformer	ViT-CB	ConvNeXt	BrainDx	SPiKe-Med proposed
Dice score	0.9278 ± 0.021	0.9397 ± 0.018	0.9124 ± 0.024	0.9486 ± 0.015	0.9728 ± 0.010
IoU score	0.9114 ± 0.022	0.9248 ± 0.019	0.8945 ± 0.025	0.9342 ± 0.016	0.9584 ± 0.011
Hausdorff distance	4.18 ± 0.45	3.76 ± 0.40	4.82 ± 0.54	3.28 ± 0.32	2.11 ± 0.21
Energy per inference	0.77 ± 0.044	0.70 ± 0.039	0.84 ± 0.051	0.66 ± 0.033	0.41 ± 0.021
Spike-rate	0.027 ± 0.0023	0.023 ± 0.0020	0.031 ± 0.0026	0.020 ± 0.0017	0.011 ± 0.0010
BCE loss	0.122 ± 0.012	0.104 ± 0.010	0.142 ± 0.014	0.094 ± 0.009	0.051 ± 0.005
Pixel accuracy	0.9665 ± 0.013	0.9738 ± 0.011	0.9582 ± 0.015	0.9789 ± 0.009	0.9926 ± 0.005
Precision	0.9162 ± 0.021	0.9285 ± 0.018	0.9012 ± 0.024	0.9374 ± 0.015	0.9639 ± 0.010
Sensitivity	0.9104 ± 0.022	0.9226 ± 0.019	0.8941 ± 0.025	0.9328 ± 0.016	0.9591 ± 0.011
Specificity	0.9291 ± 0.020	0.9412 ± 0.017	0.9185 ± 0.022	0.9495 ± 0.014	0.9752 ± 0.009
F-measure	0.9203 ± 0.021	0.9328 ± 0.018	0.9062 ± 0.023	0.9401 ± 0.015	0.9671 ± 0.010
MCC	0.9002 ± 0.023	0.9169 ± 0.020	0.8821 ± 0.026	0.9286 ± 0.017	0.9554 ± 0.011

Table 6 shows the comparative performance of the proposed SPiKe-Med framework and other models using the CT dataset. Results are shown as the average ± standard deviation for multiple experimental trials. SPiKe-Med obtained the best Dice Score (0.9812 ± 0.009) and IoU Score (0.9685 ± 0.010), which shows that the proposed framework obtained superior segmentation results than the current frameworks. Additionally, SPiKe-Med had the minimum Energy per Inference value (0.33 ± 0.017), showing increased computational efficiency. The proposed model outperformed the others using the rest of the evaluation metrics as well; namely, boundary accuracy, pixel accuracy, precision, sensitivity, specificity, F-measure, and MCC. Additionally, the small standard deviations show consistency of the proposed model in multiple experimental trials.

**Table 6:** Comparative performance of existing models on the CT dataset.

Metric	Gatransformer	ViT-CB	ConvNeXt	BrainDx	SPiKe-Med proposed
Dice score	0.9332 ± 0.019	0.9469 ± 0.016	0.9186 ± 0.022	0.9569 ± 0.014	0.9812 ± 0.009
IoU score	0.9184 ± 0.021	0.9324 ± 0.018	0.9024 ± 0.024	0.9431 ± 0.015	0.9685 ± 0.010
Hausdorff distance	3.96 ± 0.42	3.41 ± 0.37	4.56 ± 0.51	2.96 ± 0.29	1.74 ± 0.18
Energy per inference	0.73 ± 0.041	0.66 ± 0.036	0.81 ± 0.049	0.59 ± 0.031	0.33 ± 0.017
Spike-rate	0.024 ± 0.0021	0.020 ± 0.0018	0.029 ± 0.0024	0.017 ± 0.0015	0.008 ± 0.0008
BCE loss	0.114 ± 0.011	0.093 ± 0.009	0.134 ± 0.013	0.082 ± 0.008	0.039 ± 0.004
Pixel accuracy	0.9705 ± 0.012	0.9781 ± 0.010	0.9621 ± 0.014	0.9832 ± 0.008	0.9951 ± 0.004
Precision	0.9221 ± 0.020	0.9354 ± 0.017	0.9085 ± 0.023	0.9462 ± 0.014	0.9726 ± 0.009
Sensitivity	0.9176 ± 0.021	0.9306 ± 0.018	0.9012 ± 0.024	0.9418 ± 0.015	0.9694 ± 0.010
Specificity	0.9368 ± 0.018	0.9478 ± 0.016	0.9228 ± 0.020	0.9584 ± 0.013	0.9838 ± 0.008
F-measure	0.9269 ± 0.019	0.9392 ± 0.017	0.9114 ± 0.022	0.9496 ± 0.014	0.9761 ± 0.009
MCC	0.9078 ± 0.022	0.9246 ± 0.019	0.8897 ± 0.025	0.9371 ± 0.016	0.9648 ± 0.010

**Figure 12:**
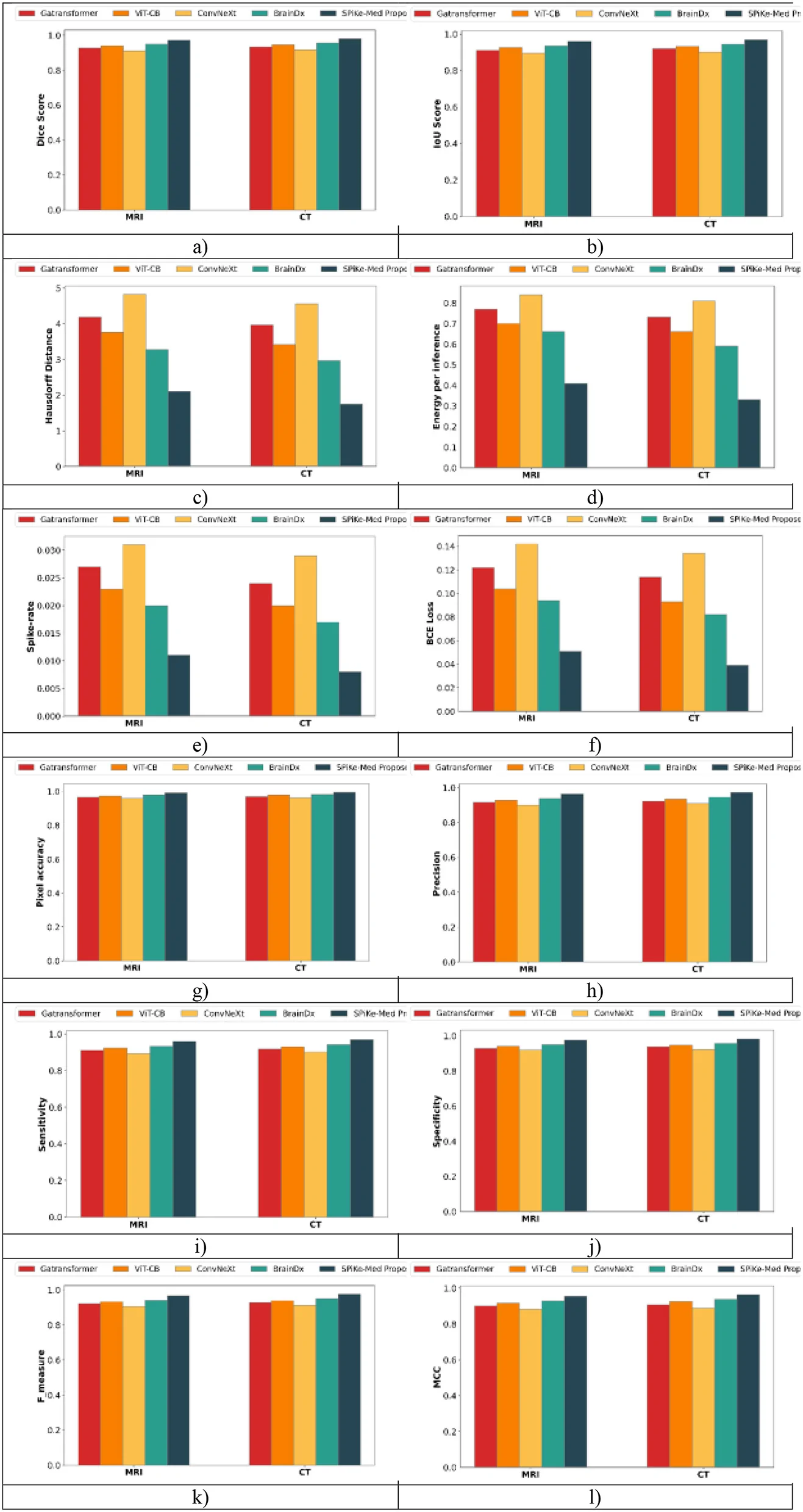
**(a)** Dice score, **(b)** IoU score, **(c)** Hausdorff distance, **(d)** Energy per inference, **(e)** Spike rate, **(f)** BCE loss, **(g)** Pixel accuracy, **(h)** Precision, **(i)** Sensitivity, **(j)** Specificity, **(k)** F-measure, **(l)** MCC.

The performance comparison between the proposed SPiKe-Med and the existing methods using MRI and CT images is shown in Figure 12. It is clearly seen from the visualization that the SPiKe-Med framework performs better than the existing algorithms with respect to all evaluated metrics of performance. With respect to the existing algorithms, the SPiKe-Med framework has higher segmentation efficacy, with increased accuracy-based metrics values, whereas it shows lower boundary deviation and energy utilization at the same time. The comparable performance of the SPiKe-Med framework for the two kinds of images (MRI and CT) suggests that the SPiKe-Med framework shows stable performance irrespective of image type. In addition, the lower variance in the results of the evaluation shows better reliability of the proposed method than the existing algorithms.

**Figure 13:**
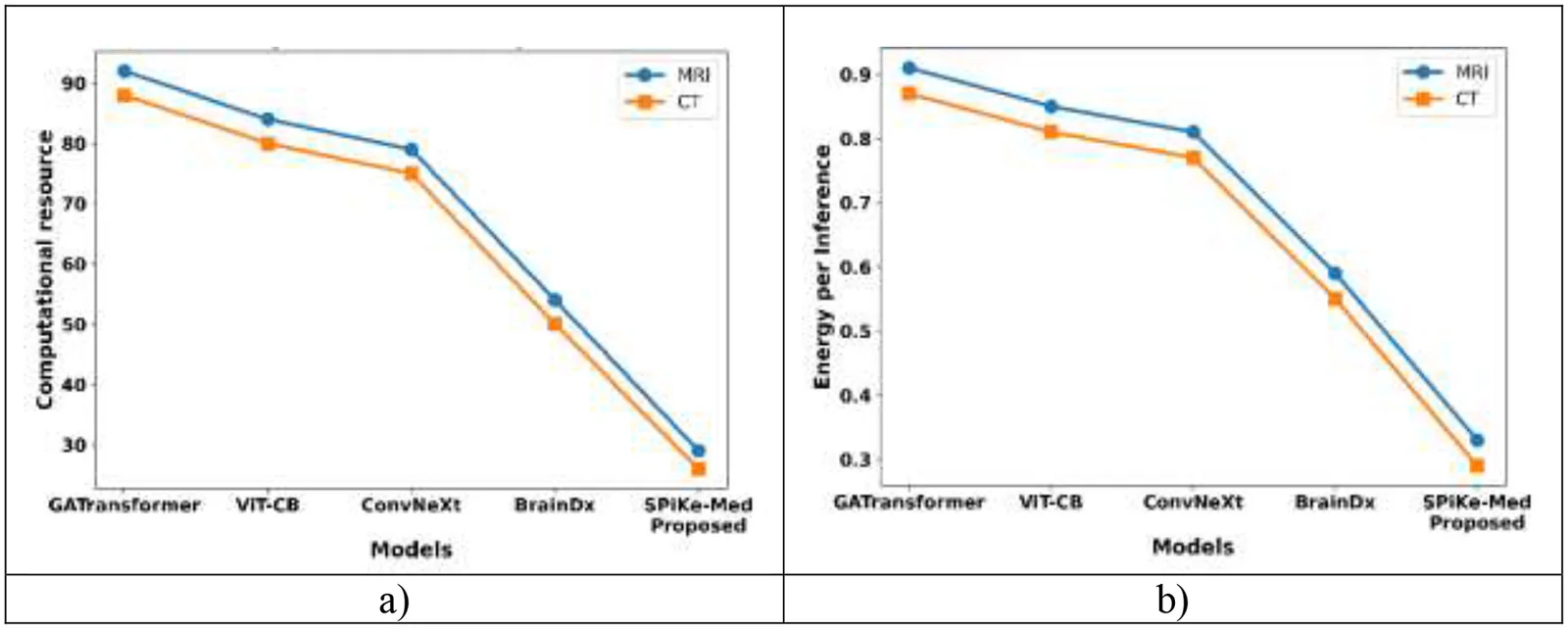
**(a)** Computational resources using both datasets, **(b)** Inference speed using both datasets.

As indicated in Figure 13 and Table 7, SPiKe-Med always uses the least number of computational resources and has the highest inference speed, both with the MRI dataset and the CT dataset. SPiKe-Med is lower in GFLOPs than existing models by over 40%−75% and has lower latency per sample. These findings validate the fact that the proposed hybrid architecture is computationally efficient and suitable for resource-constrained and near-real-time clinical applications.

**Table 7:** Computational resource and inference speed comparison across models.

Model	MRI dataset		CT dataset	
	Computational resources (GFLOPs)	Inference speed (ms/sample)	Computational resources (GFLOPs)	Inference speed (ms/sample)
Gatransformer	16.8	24	18.9	28
ViT-CB	13.5	20	15.7	23
ConvNeXt	11.2	18	12.8	21
BrainDx	9.6	16	10.9	19
Proposed	7.4	11	8.1	13

Table 8 summarizes the results of ablation analysis of our proposed SPiKe-Med framework on the MRI dataset. As expected, all the structural elements play important roles in the segmentation performance and computational efficiency of the whole network. In the absence of any architectural elements (SNN refinement module, transformer backbone, temporal consistency loss or attention residual block), the model suffered from decreases in Dice score and IoU score and increases in boundary error, energy consumption, and spike activities. The SPiKe-Med with fully integrated transformer and neuromorphic learning achieved the optimal segmentation accuracy, with Dice score and IoU score being 0.9728 and 0.9584, respectively.

**Table 8:** Ablation study of the proposed model using the MRI dataset.

Metrics	Dice score	IoU score	Hausdorff distance	Energy per inference	Spike-rate	BCE loss
Without SNN refinement	0.9512	0.9286	3.84	0.73	0.024	0.094
Without transformer backbone	0.9587	0.9365	3.26	0.65	0.02	0.081
Without temporal consistency loss	0.9643	0.9448	2.74	0.54	0.016	0.067
Without attention residual block	0.9681	0.9512	2.39	0.47	0.013	0.058
SPiKe-Med	0.9728	0.9584	2.11	0.41	0.011	0.051

Table 9 gives an ablation study of the developed SPiKe-Med framework on the CT dataset. The data reveals that all of the parts play a critical role in terms of accuracy and energy efficiency. Elimination of the SNN refinement stage, transformer backbone, temporal consistency loss or attention residual block resulted in reduced Dice and IoU performance and increased boundary error, energy consumption and spike activity. Overall performance of the whole SPiKe-Med framework was the highest with a Dice Score of 0.9812 and IoU Score of 0.9685, proving the viability of a combination of transformer and neuromorphic refinement techniques for CT brain tumor segmentation.

**Table 9:** Ablation study of the proposed model using the CT dataset.

Metrics	Dice score	IoU score	Hausdorff distance	Energy per inference	Spike-rate	BCE loss
Without SNN refinement	0.9624	0.9417	3.08	0.61	0.019	0.072
Without transformer backbone	0.9681	0.9496	2.64	0.53	0.015	0.061
Without temporal consistency loss	0.9735	0.9562	2.18	0.44	0.012	0.052
Without attention residual block	0.9772	0.9614	1.92	0.38	0.01	0.045
SPiKe-Med	0.9812	0.9685	1.74	0.33	0.008	0.039

Table 10 displays the five-fold cross-validation performance for the proposed SPiKe-Med framework on the MRI data set. The segmentation performance was not only high across the validation folds, but was also computationally stable across different validation folds. The standard deviation for all metrics was very small, signifying stable learning ability. The Dice Score and IoU Score of the SPiKe-Med were 0.9725, 0.0028, and 0.9580, 0.0040, respectively, with low energy consumption, low spike occurrence and stable loss.

**Table 10:** Five-fold cross-validation of the proposed model using the MRI dataset.

Fold	Fold-1	Fold-2	Fold-3	Fold-4	Fold-5
Dice score	0.9684	0.9709	0.9728	0.9741	0.9762
IoU score	0.9521	0.9556	0.9584	0.9603	0.9637
Hausdorff distance	2.26	2.18	2.11	2.05	1.98
Energy per inference	0.44	0.42	0.41	0.39	0.37
Spike-rate	0.013	0.012	0.011	0.01	0.009
BCE loss	0.058	0.054	0.051	0.048	0.045

SPiKe-Med is evaluated using five-fold cross-validation, and the proposed method applied on CT data is summarized in Table 11. This shows that the proposed framework performs well on segmentations with consistent accuracy and is computationally stable while reliably generalizing on each cross-validation set. As observed in the results of five-fold cross-validation, it achieved a mean Dice Score of 0.9809 0.0026 and IoU Score of 0.9681 0.0037 with few boundary errors, minimum energy and spike cost and stable convergence of the energy function for all folds.

**Table 11:** Five-fold cross-validation of the proposed model using the CT dataset.

Fold	Fold-1	Fold-2	Fold-3	Fold-4	Fold-5
Dice score	0.9774	0.9791	0.9812	0.9828	0.9841
IoU score	0.9632	0.9654	0.9685	0.9707	0.9729
Hausdorff distance	1.89	1.82	1.74	1.68	1.61
Energy per inference	0.36	0.35	0.33	0.32	0.31
Spike-rate	0.01	0.009	0.008	0.007	0.006
BCE loss	0.044	0.042	0.039	0.036	0.034

Table 12 summarizes the results from statistical significance testing of SPiKe-Med against existing approaches using the MRI dataset. The resulting findings have shown that there is a statistically significant difference between SPiKe-Med and the other approaches, as all the *p*-values were lower than the significance level (α = 0.05). The negative values of t and Wilcoxon Z confirm that the improvements in performance provided by SPiKe-Med are greater than those of the other approaches being compared.

**Table 12:** Statistical analysis of the proposed model using MRI dataset.

Comparison	Paired *t*-value	*t*-test *p*-value	Wilcoxon *Z*-value	Wilcoxon *p*-value	Significant (α = 0.05)
Gatransformer vs. SPiKe-Med	−8.47	<0.001	−3.84	<0.001	Yes
ViT-CB vs. SPiKe-Med	−4.18	0.003	−2.97	0.003	Yes
ConvNeXt vs. SPiKe-Med	−7.56	<0.001	−3.62	<0.001	Yes
BrainDx vs. SPiKe-Med	−2.86	0.021	−2.31	0.021	Yes

Statistical significance analysis of the SPiKe-Med model on the CT dataset is provided in Table 13. From the table, it is seen that there were statistically significant improvements made by the SPiKe-Med model compared to all existing methods, as all *p*-values were less than the significance level (α = 0.05). The constant significance obtained through both parametric and non-parametric tests assures us of the reliability of the performance improvement made by SPiKe-Med.

**Table 13:** Statistical analysis of the proposed model using CT dataset.

Comparison	Paired *t*-value	*t*-test *p*-value	Wilcoxon *Z*-value	Wilcoxon *p*-value	Significant (α = 0.05)
Gatransformer vs. SPiKe-Med	−9.12	<0.001	−4.06	<0.001	Yes
ViT-CB vs. SPiKe-Med	−4.73	0.002	−3.11	0.002	Yes
ConvNeXt vs. SPiKe-Med	−8.05	<0.001	−3.81	<0.001	Yes
BrainDx vs. SPiKe-Med	−3.24	0.012	−2.58	0.01	Yes

### 4.4 Discussion

The performance of SPiKe-Med is attributed to the combined capabilities of the hybrid model structure. The first component of the model that is the CNN layer, effectively extracts low-level features and the textures that play a major role in the detection of tumor borders. These features are then enhanced by the Swin Transformer backbone, which models the long-range spatial dependencies due to the shifted-window self-attention, thereby increasing the contextual understanding of complex tumor shapes. Contrary to regular CNN architectures, the transformer allows for better modeling of global relationships due to its ability to effectively represent global contexts. The use of a spiking neural network contributes to the next refinement step due to the temporal spike coding and sparse event-driven computations. The Leaky Integrate-and-Fire neurons filter informative feature activations and prevent any redundant responses, thus resulting in low spike activity and decreased computational complexity. The combination of Dice loss, Focal loss, and temporal consistency loss in the composite loss function additionally improves the optimization due to increased segmentation overlap, management of class imbalance and temporal consistency in spike dynamics. All these factors contribute to the improvements in segmentation accuracy, border localization, inference speed and energy consumption demonstrated.

Hybrid medical image segmentation models recently reported mainly consider the use of CNNs (Tehsin et al., 2025; Tanone et al., 2025; Lopez-Ramirez et al., 2026; Kaur et al., 2026) along with transformers to augment the feature representation by leveraging the combination of local feature extraction through CNNs and global attention mechanisms through transformers. Despite the considerable improvement in segmentation performance of such hybrid models, these models normally employ conventional dense neural computation at all stages of inference, which makes the computation complexity and energy cost relatively large. Different from these recent hybrid architectures for image segmentation, the SPiKe-Med framework considers the application of neuromorphic refinement through spike-based encoding and Leaky Integrate-and-Fire neuron models. Without substituting the traditional neural modules for CNNs or transformers, the novel architecture employs a spike-driven refinement step after hierarchical feature extraction to make event-based computation possible while maintaining segmentation performance. In addition, by using surrogate gradient learning, partial ANN-to-SNN conversion, and temperature-scaled training, the presented framework makes the three goals of image segmentation possible under one framework.

In terms of clinical applications, an effective and computationally efficient brain tumor segmentation helps radiologists in the processes of diagnosis, therapy planning, and follow-up since it provides an objective and reproducible way of segmenting tumors with less need for manual assistance. In addition, the low computation costs and low energy consumption of the SPiKe-Med allow the framework to be used in a setting where hardware resources are limited. On the other hand, there are still more tests to be done before it is applied in practice. Even in light of these promising results, several limitations need to be mentioned. The introduced framework was tested with the help of publicly available benchmark datasets; hence, it is important to conduct additional experiments based on multi-center large-scale datasets to study the generalization ability of the framework. Also, although the neuromorphic fine-tuning leads to more efficient inference, the current version of the framework still uses GPU simulations, and not neuromorphic hardware. Further research will be aimed at testing the introduced framework at multi-center clinical level and on neuromorphic devices.

## 5 Conclusion

The proposed SPiKe-Med framework has managed to integrate spiking neural networks, convolutional neural networks, and transformer architectures to deliver precise and energy-efficient brain tumor segmentation. The model balances precision, computational efficiency and robustness through the incorporation of neuromorphic-inspired processing with advanced DL backbones. The multi-scale nature of frameworks and the time-encoding processes of segments improve the quality of segmentation; they also minimize energy usage and latency. On the whole, SPiKe-Med proves to be a promising, scalable and reliable solution to real-time medical image analysis. Clinically, SPiKe-Med balances high segmentation accuracy and computational efficiency. Its spiking refinement stage allows sparse, event-based inference with low energy usage and low latency that is maintained.

## Data Availability

The original contributions presented in the study are included in the article/supplementary material, further inquiries can be directed to the corresponding author.
